# Food-Associated Xenobiotics and Innate Immunity: Molecular and Cellular Effects of Imazalil, TBHQ (Tert-Butylhydroquinone), Carrageenans, and Monosodium Glutamate

**DOI:** 10.3390/ijms27219580

**Published:** 2026-10-26

**Authors:** Fatemeh Sayareh, Grzegorz Zwierzchowski, Roman Wójcik

**Affiliations:** 1 Department of Microbiology and Clinical Immunology, Faculty of Veterinary Medicine, University of Warmia and Mazury, 10-719 Olsztyn, Poland; fatemeh.sayareh@doktorant.uwm.edu.pl; 2 Department of Biochemistry, Faculty of Biology and Biotechnology, University of Warmia and Mazury, 10-719 Olsztyn, Poland; grzegorz.zwierzchowski@uwm.edu.pl

**Keywords:** food additives, dietary xenobiotics, innate immunity, TBHQ, imazalil, carrageenans, monosodium glutamate, inflammation, oxidative stress, microbiota

## Abstract

Food-associated xenobiotics may influence innate immune responses, but the relevance of experimental findings to human dietary exposure remains uncertain. This narrative review critically examines evidence concerning imazalil, tert-butylhydroquinone (TBHQ), carrageenans, and monosodium glutamate (MSG), representing a post-harvest fungicide, a synthetic antioxidant, a hydrocolloid, and a flavor enhancer, respectively. Experimental findings are compared across cellular and humoral innate immune endpoints, with attention to chemical form, dose, administration route, exposure duration, and the distinction between biomarker changes and adverse functional outcomes. Reported effects involve redox-sensitive signaling, cytokine regulation, macrophage and neutrophil responses, NK-cell activation and effector-associated markers, and intestinal barrier–microbiota interactions. These overlapping processes do not establish equivalent mechanisms or immunotoxic potential. TBHQ has a comparatively well-characterized connection to Nrf2 activation, although Nrf2 dependence has not been established for every reported immune effect. Imazalil evidence is narrower and requires independent replication, whereas findings for carrageenans depend strongly on material characterization and experimental context. MSG findings vary with dose, route, life stage, and metabolic conditions. Moreover, the predominantly preclinical literature includes exposure conditions whose correspondence to human dietary intake is uncertain. Consequently, available evidence supports context-dependent innate immune modulation but does not establish clinically meaningful immune impairment at customary dietary exposure. Further progress requires exposure-informed studies linking molecular and cellular changes to persistent functional effects and adverse outcomes, supported by human-relevant systems and human investigations where feasible. Such evidence would clarify the contribution of innate immune endpoints to food-safety assessment.

## 1. Introduction

Food additives, including antioxidants, stabilizers, thickeners, and flavor enhancers, are widely used to preserve food quality, improve sensory properties, and maintain product stability [1,2]. Together with pesticide residues, they contribute to dietary exposure to chemically diverse substances whose biological effects depend on their properties and exposure conditions. Although food-safety assessment encompasses established toxicological endpoints, the effects of repeated dietary exposure on immune function remain incompletely characterized [3,4]. Consequently, evaluating immune-related findings requires consideration of both their biological significance and the exposure conditions under which they occur.

Innate immunity provides a relevant framework for this assessment because it integrates soluble mediators and cellular responses involved in pathogen defense and tissue homeostasis [5,6,7]. Changes in cytokine production, inflammatory signaling, or immune-cell phenotype may therefore indicate a response to chemical exposure. However, these changes do not independently establish an adverse immune effect [3,8]. Their interpretation should also incorporate functional endpoints, including phagocytosis, leukocyte migration, natural killer (NK) cell-mediated killing, and, where available, host resistance to infection [9]. Within this framework, redox-sensitive signaling and barrier–microbiota interactions may help explain how food-associated compounds influence innate responses, provided that mechanistic changes are distinguished from their functional consequences [1,10].

Against this background, the present narrative review examines imazalil (IMZ), tert-butylhydroquinone (TBHQ), κ-carrageenan (κ-CGN), and monosodium glutamate (MSG). These compounds were selected to compare distinct categories of food-associated exposure—a post-harvest fungicide residue, a synthetic antioxidant, a hydrocolloid, and a flavor enhancer—and contrasting experimental evidence concerning innate immune responses. They do not constitute a representative sample of all food additives or dietary contaminants. Despite their chemical and functional differences, each has been investigated in relation to processes relevant to innate immunity. Imazalil studies have reported changes in neutrophil function, intestinal barrier homeostasis, microbiota composition, and cytokine expression [11,12,13,14]. TBHQ, in turn, has been studied as an activator of nuclear factor erythroid 2-related factor 2 (Nrf2) and a modulator of immune-cell activation [15,16]. Carrageenan-associated inflammatory and mucosal effects depend strongly on molecular form and experimental context [17,18,19], whereas MSG studies have described changes in immune-cell populations, inflammatory mediators, and redox balance in preclinical models [20,21]. Taken together, these findings provide a basis for comparing innate immune responses across distinct chemical classes, while retaining the conditions and limitations specific to each compound.

The aim of this review is therefore to critically synthesize the molecular and cellular effects of these four compounds on innate immunity, distinguishing direct functional evidence from biomarker changes and contextual findings. To support comparison, the compound-specific analyses follow a common structure covering exposure characteristics, innate immune cells and effector functions, soluble mediators and barrier–microbiota interactions, mechanistic interpretation, and exposure relevance. Evidence concerning adaptive immunity or non-immune toxicity is considered only where it helps explain the innate immune findings. This approach provides a basis for identifying shared mechanisms, compound-specific differences, and the principal gaps that limit conclusions about adverse immune effects under realistic dietary conditions.

## 2. Literature Selection and Interpretative Approach

### 2.1. Literature Selection

This narrative review draws on the literature retrieved from PubMed, Scopus, Web of Science, and Google Scholar using combinations of compound names (imazalil, TBHQ, carrageenans, and MSG) and terms related to innate immunity, including cytokines, nuclear factor κB (NF-κB), Nrf2, Toll-like receptor 4 (TLR4), phagocytosis, NK cell cytotoxicity, and gut microbiota. Priority was given to original experimental and mechanistic studies published within the preceding 10 years, particularly those using oral, dietary, or biologically plausible exposure conditions. However, older foundational studies were retained where they provided relevant evidence not superseded by more recent research. Studies using high experimental concentrations, non-dietary administration routes, or materials distinct from food-grade preparations were also considered, with their exposure limitations addressed alongside the findings. Searches were conducted between January and July 2026.

As this was a narrative rather than systematic review, no formal risk-of-bias scoring or quantitative synthesis was performed. Instead, the evidence was compared qualitatively in terms of consistency, mechanistic support, functional significance, model relevance, and correspondence to human dietary exposure. Accordingly, mechanistic support and dietary-exposure relevance were considered separately, since a study may clarify a cellular pathway without establishing its significance under customary dietary conditions.

Key studies informing the compound-specific conclusions were tabulated by model, tested material, dose or concentration, administration conditions, exposure duration, measured findings, and limitations affecting interpretation (Table S1). Distinct experimental arms were separated where material characteristics or administration conditions differed. Relevant null findings were retained, and mechanistic support was considered separately from relevance to human dietary exposure.

Conference abstracts were identified as preliminary evidence and interpreted within the limits of the available methodological information. They were not treated as equivalent to full experimental reports.

### 2.2. Interpretation of Immune-Related Endpoints

The interpretation of immune-related findings distinguishes general cellular stress, inflammation, immunomodulation, and immunotoxicity. General cellular stress encompasses responses such as oxidative imbalance, endoplasmic reticulum stress, and mitochondrial dysfunction that are not specific to immune cells. Inflammation involves coordinated cellular and mediator responses that may be protective or contribute to pathology, whereas immunomodulation denotes a measurable change in immune activity without necessarily implying harm. Immunotoxicity, in contrast, refers to adverse effects on immune structure or function, including impaired host defense or immune-mediated tissue injury [3,8,9].

Within this framework, cytokine profiles, acute-phase proteins, and redox-related markers provide evidence of biological responses but do not independently establish adversity. Their significance depends on the tissue examined, sampling time, magnitude and persistence of the response, exposure conditions, and association with functional or pathological outcomes [3,8,21,22]. Consequently, biomarker changes are interpreted alongside immune-cell function and tissue-level findings rather than treated as equivalent evidence of immunotoxicity [3,23,24].

Functional endpoints include phagocytosis, chemotaxis, oxidative burst, intracellular killing, and NK cell-mediated target-cell killing [23,25,26,27,28]. These measurements provide information that cannot be inferred from leukocyte counts or activation markers alone. Nevertheless, individual assays address distinct components of effector function: phagocytosis does not establish microbial killing, and NK-cell degranulation or expression of cytolytic proteins does not directly demonstrate target-cell death [23,25,29,30]. Where available, bacterial clearance and infection-challenge outcomes therefore provide complementary evidence of consequences for host defense.

Functional findings also require consideration of cell viability, cell source, stimulation conditions, and assay variability [31,32,33]. Accordingly, reduced activity associated with cytotoxicity is distinguished from functional alteration in viable cells, while changes in reactive oxygen species (ROS) production or inflammatory signaling are interpreted in relation to the biological context.

### 2.3. Relevance to Human Dietary Exposure

Exposure relevance is considered in relation to the identity and characteristics of the tested material, dose or concentration, administration route, vehicle or food matrix, exposure duration, and host condition. In particular, dietary feeding, drinking-water administration, and intragastric gavage are treated as distinct exposure scenarios. Although gavage is an oral route, it delivers a bolus and does not reproduce the food-matrix context of customary consumption. Likewise, native food-grade carrageenan is distinguished from degraded preparations, oligosaccharides, and chemically modified derivatives.

For in vitro and ex vivo studies, nominal concentrations are not equated directly with dietary intake, because absorption, metabolism, and tissue distribution determine the exposure reaching immune cells. Such studies are therefore interpreted primarily as mechanistic evidence unless a plausible relationship to internal or intestinal exposure can be established. Similarly, regulatory intake limits provide reference points for exposure assessment rather than direct equivalents of cellular concentrations.

Taken together, these considerations allow the review to distinguish evidence of a biological mechanism, an adverse immune effect under experimental conditions, and a potential risk at human dietary exposure levels. They also provide a common basis for interpreting the four compounds without assuming that comparable biomarker changes imply equivalent functional consequences or consumer risk.

## 3. Overview of Innate Humoral and Cellular Immunity

Innate immunity integrates soluble mediators, cellular effector mechanisms, and epithelial defenses to contain infection and maintain tissue homeostasis [5,6,7,25]. Its humoral components include cytokines, chemokines, complement proteins, and acute-phase proteins, which coordinate inflammatory responses and support pathogen recognition and clearance [34,35]. Cytokines regulate leukocyte activation, while chemokines direct cell recruitment to affected tissues. Complement further connects humoral and cellular defense through opsonization, inflammatory signaling, and the lysis of susceptible microorganisms [36]. In the literature examined here, humoral findings predominantly concern cytokines, chemokines, and selected acute-phase proteins; direct assessments of complement and other specialized soluble defense mechanisms are comparatively limited.

The cellular components most relevant to this review are macrophages, neutrophils, and NK cells. Macrophages and neutrophils participate in microbial recognition, phagocytosis, and antimicrobial responses involving ROS and granule-associated products [26,27]. Their production of inflammatory mediators also influences the recruitment and activity of other immune cells. NK cells contribute to early defense against infected or transformed cells through cytotoxic mechanisms and cytokine secretion [28,37], while dendritic cells link innate recognition to subsequent adaptive responses [38]. These complementary activities provide the biological context for the cellular endpoints examined in the compound-specific sections.

Humoral and cellular responses are closely connected: soluble mediators regulate leukocyte behavior, and activated cells modify the surrounding mediator environment [35,39]. At intestinal surfaces, these interactions also involve epithelial integrity and signals originating from the microbiota. The following sections examine how imazalil, TBHQ, carrageenans, and MSG have been investigated in relation to this network, focusing on inflammatory mediators, phagocyte and NK-cell responses, redox-sensitive signaling, and barrier–microbiota interactions. This framework organizes the biological targets considered in the review; the criteria for interpreting experimental findings and their dietary relevance are described in the preceding section.

## 4. Imazalil and Innate Immune Modulation

### 4.1. Dietary Exposure and Characteristics Relevant to Interpretation

Imazalil (IMZ) is a synthetic imidazole fungicide belonging to the conazole class, with protective and curative activity against fungal diseases [14,40]. Its agricultural applications include post-harvest treatment of citrus fruits, apples, and pears, as well as seed treatment and other crop-protection uses [40,41,42,43,44]. Consequently, its relevance to dietary immunotoxicology arises primarily from residues in treated food rather than intentional use as a food additive. IMZ is also chiral and is commonly supplied as a racemic mixture; its lipophilicity and moderate water solubility are relevant to the interpretation of experimental preparations and internal exposure [14,45].

A recent human neutrophil study selected 0.07 ng/mL as its lowest experimental concentration on the basis of an earlier report of IMZ detection in human serum [14]. This provides a link to measured internal exposure, although it does not establish that this concentration is typical of consumers or that short-term exposure of cells in culture reproduces chronic dietary exposure. Conversely, administered doses in animal studies describe external exposure and cannot be compared directly with cell-culture concentrations without toxicokinetic information.

The principal intestinal studies also differ substantially in exposure conditions. In the initial study, male ICR mice received IMZ mixed into chow at reported doses of 25, 50, or 100 mg/kg body weight/day for 28 days [11]. A subsequent study used male C57BL/6 mice exposed orally to 0.1, 0.5, or 2.5 mg/kg body weight/day for 2, 5, or 15 weeks [13]. In the maternal-exposure study, pregnant C57BL/6 mice were treated during gestation and lactation at levels estimated by the authors to deliver approximately 5 or 50 mg/kg body weight/day [12].

### 4.2. Effects on Innate Immune Cells and Effector Functions

Direct evidence concerning the effects of IMZ on innate immune cell function remains limited to a human ex vivo study among the experimental papers examined here. In neutrophils obtained from 15 healthy male donors, short-term exposure to 0.07 and 0.7 ng/mL reduced latex particle uptake, whereas chemotaxis was impaired only at 0.7 ng/mL; neither endpoint was significantly altered at 7 ng/mL [14]. These findings indicate changes in two complementary components of neutrophil defense—migration toward a stimulus and engulfment of particulate targets. However, because the experiments did not assess microbial killing or recruitment in vivo, they establish altered cellular functions under the tested conditions rather than impaired resistance to infection.

The accompanying oxidative responses suggest that these effects cannot be described as uniform suppression of neutrophil activity. At 0.7 ng/mL, IMZ increased spontaneous nitroblue tetrazolium (NBT) reduction without significantly changing the latex-stimulated response, while nitric oxide production increased at 7 ng/mL. Meanwhile, viability remained approximately 98% after two hours at all tested concentrations, making overt cytotoxicity an unlikely explanation for the observed functional changes [14]. Taken together, these findings indicate divergent effects on neutrophil effector processes, with reduced particle uptake or migration accompanied by increased spontaneous oxidative activity. Nevertheless, the concentration-dependent differences require replication and a broader concentration range before a general non-monotonic response can be established.

### 4.3. Innate Immune Mediators and Barrier–Microbiota Interactions

Animal studies extend this evidence to intestinal inflammation and epithelial components of mucosal defense. After 28 days of dietary exposure to 25–100 mg/kg body weight/day, male ICR mice exhibited changes in gut microbial composition and colonic inflammatory indicators [11]. Inflammatory-cell coverage increased at 50 and 100 mg/kg body weight/day, while fecal lipocalin-2 increased at the highest dose after 21 and 28 days. Colonic transcripts of TNF-α, IL-1β, IL-22, and IFN-γ were elevated across the treated groups; IL-6 transcription also increased at the highest dose [11]. These findings support an intestinal inflammatory response, although transcript abundance should be distinguished from cytokine protein concentrations and the tissue-level measurements do not identify the producing cells.

The withdrawal phase further qualifies the interpretation. Following cessation of the highest-dose exposure, fecal lipocalin-2 returned to control levels by approximately seven days, whereas recovery of microbial composition differed among bacterial groups. Firmicutes and Actinobacteria returned to control levels rapidly, but the relative abundance of Bacteroidetes remained reduced during the 35-day observation period [11]. Consequently, persistent changes in selected microbial groups should not be equated with persistent inflammation.

At lower administered doses and longer exposure durations, the C57BL/6 study documented changes in microbial composition, mucus secretion, and the expression or abundance of proteins associated with epithelial ion transport [13]. These endpoints are relevant to mucosal homeostasis, but they do not constitute a direct measurement of intestinal permeability. Moreover, recovery was tissue-dependent: after 45 days without IMZ, the measured ileal transporter protein levels returned to control values, whereas corresponding abnormalities in the colon persisted. Several microbial differences also resolved during withdrawal [13]. The evidence therefore indicates a mixture of reversible and persistent changes rather than uniformly sustained barrier impairment.

Maternal exposure produced additional changes in mucus-associated endpoints, ion transporters, antimicrobial peptide transcripts, and bile acid metabolism in dams and their F1 and F2 offspring [12]. However, antimicrobial peptide responses varied with sex, age, generation, and exposure level, including both increases and decreases. Accordingly, these results should be described as altered regulation of epithelial defense rather than a consistent suppression of antimicrobial peptide expression.

A prominent finding was increased IL-22 mRNA in liver and ileum across the examined generations and ages [12]. IL-22 can support epithelial repair and antimicrobial defense and can be produced by both innate lymphoid cells and T-cell subsets [46]. Nevertheless, the IMZ study did not identify its cellular source or establish whether the increased transcription represented protective compensation, inflammatory activation, or both. Positive correlations with bile acid changes suggest a relationship between metabolic disturbance and cytokine regulation, but do not establish causality [12]. Evidence linking IL-22-associated pathways to protection against enteric pathogens provides relevant biological context [47]; it does not demonstrate that the IMZ-associated response improved resistance to infection.

### 4.4. Mechanistic Interpretation

The available findings support two potentially complementary routes of immune modulation: effects on leukocyte effector processes and changes in the intestinal environment that may influence mucosal immune regulation [11,12,13,14]. The human cell study demonstrates functional changes in the absence of an intact gut–liver system, whereas the mouse studies connect exposure with microbial, epithelial, inflammatory, and metabolic endpoints. Nevertheless, neither evidence stream establishes a complete causal sequence from IMZ exposure to impaired host defense.

In particular, simultaneous changes in microbiota and epithelial endpoints do not determine which occurred first. Microbial shifts could influence epithelial and immune regulation, while altered mucus secretion or intestinal transport could themselves change microbial habitats. Likewise, molecular docking in the mouse intestinal study suggested possible interactions with epithelial transport proteins, but did not demonstrate binding in vivo or inhibition of transport activity [13]. These mechanisms should therefore remain hypotheses rather than experimentally established explanations.

The antifungal action of IMZ involves inhibition of fungal sterol 14α-demethylase (CYP51) and disruption of ergosterol biosynthesis [48]. However, this mechanism is distinct from its effects on mammalian steroid-related pathways. Endocrine and metabolic effects provide further context. IMZ-associated changes in steroid-related pathways have been reported in maternal-exposure and developmental models, while experimental work has examined its interference with aromatase-dependent estrogen synthesis [49,50,51]. Such findings may be relevant because sex hormones participate in innate immune regulation [52]. However, these mammalian effects should be distinguished from the compound’s antifungal action, and they do not establish that endocrine disruption caused the neutrophil or intestinal immune changes. Similarly, hepatotoxicity findings may help interpret disturbances along the gut–liver axis, but remain contextual evidence rather than direct measures of innate immune dysfunction [53].

Epigenetic observations also require separation from claims of inherited immunotoxicity. Studies reporting altered hepatic mRNA methylation in dams and offspring, or epigenetic and transcriptional changes in *Xenopus*, suggest molecular responses to IMZ exposure [54,55]. They do not, however, demonstrate that these changes mediate inherited alterations in innate immunity. In the maternal study, gestational exposure of F0 could also expose F1 fetuses and their developing germ cells, which contribute to F2. Consequently, findings in F0–F2 support multigenerational effects but do not establish transgenerational inheritance [12].

### 4.5. Evidence Limitations and Relevance to Human Dietary Exposure

The evidence for IMZ-associated innate immune modulation combines one human ex vivo functional study with related mouse studies addressing intestinal inflammation and mucosal homeostasis [11,12,13,14]. Although the mouse studies encompass different strains, exposure regimens, and experimental designs, their origin within the same research group limits the independence of the available evidence.

Exposure relevance remains a central limitation. The human study used a concentration linked to a previous serum measurement, but short-term incubation cannot reproduce absorption, metabolism, protein binding, or repeated exposure in vivo [14]. Conversely, the mouse studies used oral exposure, including administration through treated chow in male ICR mice [11]. However, oral administration alone does not establish relevance to consumer exposure, and doses expressed per kilogram of body weight cannot be compared directly with residue limits expressed per kilogram of food [11,12,13].

The biological interpretation also depends on the endpoint. Reduced neutrophil particle uptake and chemotaxis constitute functional changes, whereas tissue cytokine transcripts, microbial composition, mucus staining, and transporter abundance describe different aspects of immune-associated physiology [11,12,13,14]. None alone demonstrates increased susceptibility to infection, and the studies did not establish a causal link between these changes and impaired pathogen clearance. Moreover, the withdrawal experiments show that reversibility varies by tissue and endpoint, limiting broad claims of sustained dysfunction [11,13].

Accordingly, future studies should combine exposure estimates and internal dosimetry with replicated functional assays, direct barrier measurements, and host-defense outcomes. Maternal-exposure designs should additionally distinguish direct developmental exposure from effects persisting into an unexposed generation. At present, IMZ can be described as a compound capable of modifying selected innate immune and mucosal endpoints under experimental conditions, while the consequences of typical human dietary exposure remain unresolved.

## 5. Synthetic Antioxidants and Innate Immunity: Focus on TBHQ

### 5.1. Dietary Exposure and Characteristics Relevant to Interpretation

Tert-butylhydroquinone (TBHQ, E319) is a synthetic phenolic antioxidant used to limit lipid oxidation in oils, fats, and oil-rich foods, thereby preserving product quality during storage. Its antioxidant activity involves both the parent compound and transformation products, including tert-butylbenzoquinone, which can participate in reactions that sustain antioxidant activity in food systems [56]. However, this technological function should be distinguished from its effects on redox-sensitive signaling in living cells.

Dietary consumption is the principal exposure route relevant to this review. Following absorption, TBHQ undergoes metabolism, including oxidation and conjugation, and urinary elimination of conjugated metabolites [57]. Human metabolic studies summarized in Joint FAO/WHO Expert Committee on Food Additives (JECFA) evaluations likewise describe urinary sulfate and glucuronide conjugates. Consequently, the activity of TBHQ in an experimental culture cannot be interpreted independently of the metabolism and clearance that determine exposure in vivo.

In the United States, the permitted total antioxidant content of food containing TBHQ must not exceed 0.02% of the food’s oil or fat content, equivalent to 200 mg/kg of that fraction [57,58]. This is not a universal limit of 200 mg/kg of the complete food product. JECFA established an acceptable daily intake (ADI) of 0–0.7 mg/kg body weight/day, subsequently confirmed by EFSA [59]. In its refined exposure assessment, the European Food Safety Authority (EFSA) reported that estimated intake did not exceed the ADI in the population groups examined under the refined scenarios, although estimates based on maximum permitted levels exceeded it among high-consuming toddlers and children [59].

These regulatory values address permitted use and estimated intake, whereas micromolar concentrations in cell culture describe nominal extracellular exposure. Accordingly, their comparison requires information on absorption, metabolism, protein binding, and tissue distribution; compliance with a food-use limit does not itself establish the concentrations encountered by immune cells.

### 5.2. Effects on Innate Immune Cells and Effector Functions

Experimental evidence indicates that TBHQ affects different innate immune cell populations in distinct ways. In RAW 264.7 murine macrophages, exposure to 0.1 µM increased uptake of fluorescent microspheres following lipopolysaccharide (LPS) or polyinosinic–polycytidylic acid stimulation, whereas 1 and 5 µM did not significantly alter uptake [60]. Thus, the macrophage findings do not support uniform inhibition of phagocytosis, and particle internalization should be distinguished from microbial killing.

A complementary study examined NK cells within splenocyte cultures from female C57BL/6J mice. Following treatment with 1 or 5 µM TBHQ and stimulation with phorbol 12-myristate 13-acetate and ionomycin for 24 h, NK cells exhibited reduced intracellular IFN-γ, granzyme B, and perforin, lower induction of CD25 and CD69, and changes in maturation-associated phenotypes [15]. These endpoints indicate altered activation and effector potential. Nevertheless, the study did not directly quantify target-cell killing, and the mixed-cell preparation leaves open contributions from other splenic populations.

Dietary exposure during influenza infection provides preliminary in vivo evidence. A conference abstract reported reduced NK-cell granzyme B expression in infected mice receiving TBHQ-containing feed [61]. This observation is consistent with altered cytotoxic potential, but does not independently establish reduced killing activity or prove that the effect was mediated by Nrf2.

Dendritic cells may also be affected. A separate conference abstract described female C57BL/6 mice fed an AIN-93G diet containing 0.0014% TBHQ for two weeks before influenza A infection. Dendritic cells from the lungs and mediastinal lymph nodes showed reduced MHC II and costimulatory marker expression, together with lower IL-6 and IL-12 production [62]. These findings concern the interface between innate sensing and adaptive activation; however, their implications for antigen presentation and antiviral protection require confirmation beyond the reported phenotypic and cytokine endpoints.

### 5.3. Innate Immune Mediators and Barrier–Microbiota Interactions

Changes in inflammatory mediators depend on concentration and stimulation. In activated RAW 264.7 macrophages, 5 µM TBHQ reduced nitric oxide output and IL-10 secretion. Lower concentrations increased IL-6 secretion, whereas its suppression at 5 µM occurred under LPS stimulation but not under polyinosinic–polycytidylic acid stimulation [60]. The findings therefore describe context-dependent modulation of inflammatory and regulatory mediators rather than a uniformly anti-inflammatory response.

Evidence from BV-2 microglia extends the analysis to another innate myeloid population. TBHQ pretreatment attenuated LPS-associated production of TNF-α, IL-1β, IL-6, prostaglandin E_2_, and nitric oxide [63]. Although obtained in a neuroinflammatory model, these results directly concern innate immune signaling. Their relevance to dietary exposure remains separate from their value in identifying cellular mechanisms.

Conversely, TBHQ can promote immune-cell recruitment through cytokine induction in experimental tissue models. Topical treatment of murine tumors increased NK-cell infiltration through a pathway requiring tumor-associated Nrf2 and IL-17D [64]. Related work demonstrated induction of IL-17D and CCL2 and recruitment of innate leukocytes following intraperitoneal administration [65]. The Nrf2–IL-17D relationship is also discussed in a subsequent mechanistic synthesis [66].

Importantly, increased recruitment and reduced effector-marker expression measure different processes. Their occurrence in different experimental systems is therefore not inherently contradictory. Among the studies considered here, the principal evidence concerns cellular signaling, cytokines, and tissue recruitment; it does not establish a dietary TBHQ–microbiota–barrier pathway comparable to that examined for imazalil.

### 5.4. Mechanistic Interpretation

TBHQ is widely used experimentally to activate Nrf2-associated antioxidant responses. Foundational studies demonstrated antioxidant response element activation in astrocyte-associated systems and linked TBHQ exposure to mitochondrial oxidative stress and Nrf2 activation [67,68]. These findings explain why antioxidant efficacy in food should not be equated with simple suppression of oxidative processes in cells: induction of antioxidant defenses can follow an initial redox disturbance.

The strength of evidence for Nrf2 involvement nevertheless varies between models. In BV-2 microglia, Nrf2-specific knockdown supported its contribution to TBHQ-associated suppression of p38 phosphorylation and inflammatory activation [63]. In contrast, the RAW 264.7 study documented antioxidant-associated transcriptional changes, including increased *Cbr3*, *Gstp1/3*, *Hmox1*, and *Gclm*, alongside reduced inflammatory and NF-κB-associated gene expression [60]. This supports a proposed Nrf2-related interpretation, but does not establish that Nrf2 was necessary for the observed effects.

Stronger causal evidence supports Nrf2-dependent induction of IL-17D in tumor models, where chromatin binding and gene-silencing experiments linked the transcription factor to cytokine regulation [64]. However, this relationship does not explain every effect of TBHQ. In the murine cytomegalovirus model, intraperitoneal treatment reduced weight loss and viral burden, while partial protection and CCL2 induction persisted in Nrf2-deficient animals [65]. Thus, both Nrf2-dependent and independent processes contributed to the response.

Studies of adaptive immunity provide an additional caution against assigning all TBHQ effects to Nrf2. TBHQ altered activation of primary human CD4^+^ T cells and influenced experimental allergic responses and T-helper differentiation [16,69,70]. Moreover, inhibition of selected cytokines occurred in both wild-type and Nrf2-null T cells in a comparative activator study [71]. These are contextual findings, but they reinforce the need to demonstrate pathway dependence separately for each innate immune endpoint.

Taken together, the evidence supports TBHQ-associated modulation of redox-sensitive immune pathways without a single direction of effect across models. Cell identity, stimulation, treatment timing, tissue compartment, and administration route all influence whether the measured response involves diminished activation, enhanced particle uptake, increased recruitment, or protection from inflammatory injury.

### 5.5. Evidence Limitations and Relevance to Human Dietary Exposure

The evidence base includes full experimental publications, regulatory assessments, general toxicology studies, and conference abstracts, which should contribute differently to the synthesis. Historical evaluations and rodent feeding studies provide background on systemic toxicity and dose selection [72,73] but cannot substitute for measurements of innate immune function. Similarly, transcriptomic changes in splenic CD4^+^ T cells following oral exposure concern adaptive immune biology and do not establish altered resistance to respiratory viruses [74].

Dietary experiments in zebrafish provide further contextual evidence. Wild-type and Nrf2a-mutant animals receiving a diet containing 5% TBHQ exhibited developmental and survival effects together with molecular changes [75]. Although the feeding route is relevant to exposure interpretation, the high dietary concentration and developmental context limit extrapolation to customary human intake. Immune-associated transcriptional changes in this model should also be distinguished from directly measured innate effector functions.

A conference abstract reported impaired primary and memory responses to influenza, including changes in T-cell responses and viral clearance, at an exposure described by the authors as relevant to the human diet [76]. However, the abstract did not provide the quantitative exposure information needed to independently assess that designation. It therefore offers preliminary host-defense evidence, principally in an adaptive immune context, rather than an established demonstration of innate immunotoxicity at typical consumer exposure.

Accordingly, the dietary influenza abstracts concerning NK cells and dendritic cells are retained here but explicitly identified as preliminary reports [61,62]. Their findings warrant further investigation, while full publications and sufficient exposure information are needed to assess reproducibility, pathway dependence, and relevance to consumers.

Future studies should connect internal exposure with functional outcomes, including microbial clearance, target-cell killing, and effective antigen presentation. Genetic or pharmacological tests of pathway dependence should accompany Nrf2-associated transcriptional measurements, and dietary studies should report compound intake, food matrix, exposure duration, and recovery. At present, TBHQ can modify innate immune activation and signaling under experimental conditions, but the available evidence does not establish that customary human dietary exposure impairs host defense.

## 6. Carrageenans as Food Additives and Modulators of Innate Immunity

### 6.1. Dietary Exposure and Characteristics Relevant to Interpretation

Carrageenans (CGNs) are sulfated polysaccharides extracted from red seaweeds and widely used as stabilizers, gelling agents, and thickeners in food [77]. Commercial sources include *Kappaphycus alvarezii* and *Kappaphycus striatum*, which are used in κ-carrageenan production [78,79]. Although these polymers share a galactan backbone, κ-, ι-, and λ-carrageenan differ in the number and position of sulfate groups and the presence of 3,6-anhydrogalactose [80,81]. These differences influence their physicochemical properties; for example, κ-carrageenan forms gels in the presence of potassium ions and is used in dairy products, desserts, sauces, and processed meats [82,83].

For immunotoxicological interpretation, however, subtype identification alone is insufficient. Native high-molecular-weight polymers must also be distinguished from deliberately degraded preparations, isolated oligosaccharides, and chemically modified derivatives. In particular, poligeenan is not interchangeable with food-grade carrageenan, and the term “low-molecular-weight carrageenan” does not establish that a preparation is poligeenan [84]. Accordingly, molecular-weight distribution, degradation method, sulfate content, and formulation should be considered alongside the nominal compound name.

This distinction is especially important when comparing direct cellular exposure with ingestion. Cell-culture experiments establish whether a preparation can affect exposed cells, whereas oral studies additionally involve the food matrix, gastrointestinal environment, and microbiota. Conversely, limited systemic absorption does not establish biological inertness at the intestinal surface. The following sections therefore distinguish evidence obtained with native polymers from findings involving degraded or modified preparations, while retaining the experimental context of each study.

### 6.2. Effects on Innate Immune Cells and Effector Functions

In RAW 264.7 cells, a κ-carrageenans preparation, with unresolved polymer/oligosaccharide terminology and unspecified molecular weight, increased NO and TNF-α production, neutral-red uptake, and TLR4/NF-κB-associated responses [85]. However, the material was described as a polysaccharide in the title and as an oligosaccharide in Methods, without a reported molecular weight. Its classification as a native food-grade polymer therefore remains unresolved. Neutral-red uptake is also distinct from direct particle engulfment or microbial killing and does not establish improved antimicrobial defense.

A comparative study examined κ- and λ-carrageenans from Chondrus armatus and their degradation products in murine peritoneal macrophages, both during direct incubation and after oral gavage at 100 µg/kg body weight/day for seven days [86]. In vitro, most preparations increased motility and reduced uptake of labeled E. coli particles, whereas λ-carrageenan degradation products reduced motility without affecting uptake. Following oral administration, all preparations reduced particle uptake, while the degradation products increased motility. These findings demonstrate preparation- and exposure-dependent functional changes rather than a uniform distinction between stimulatory native polymers and inhibitory degradation products.

An in vitro study using neutrophil/monocyte preparations from healthy human donor blood compared κ-, λ-, and hybrid κ/β-carrageenans with an acid-degraded κ/β derivative. Latex-particle uptake, ROS production, myeloperoxidase activity, and cell morphology depended on the preparation and the presence of LPS; some preparations attenuated LPS-induced ROS or myeloperoxidase responses, while induction of NO derivatives was not detected [87]. These results extend the evidence to human phagocyte responses but do not establish microbial killing or comparable effects following dietary exposure.

Studies of carrageenan oligosaccharides and their derivatives in tumor-bearing mice have additionally reported changes in macrophage phagocytosis and NK cell activity alongside antitumor responses [88,89]. These findings are relevant to the biological activity of the tested derivatives, but their interpretation depends on the tumor-associated immune environment and cannot be transferred directly to customary consumption of food-grade carrageenans.

Similarly, aquatic studies provide contextual evidence of altered innate immune parameters and resistance to infection [90,91]. Administration route remains important even within this evidence group: the carp study used intraperitoneal carrageenan injection rather than dietary supplementation [92]. Consequently, these experiments inform comparative immunobiology but provide limited direct evidence concerning human dietary exposure.

### 6.3. Innate Immune Mediators and Barrier–Microbiota Interactions

Intestinal barrier–microbiota interactions provide an important complement to direct immune-cell experiments. In mice receiving κ-carrageenans by oral gavage at 1.7, 8.3, or 41.7 mg/kg for 90 days, microbiota composition, microbiota-derived short-chain fatty acids, and mucus-layer thickness were altered without significant intestinal inflammation in the absence of infection [18]. Subsequent *Citrobacter rodentium* challenge aggravated inflammation and mucosal injury. Fecal-transfer experiments supported a microbiota-mediated contribution. These findings concern a prolonged gavage regimen and challenge-dependent susceptibility rather than spontaneous inflammation during ordinary dietary consumption.

The influence of administration conditions and dietary background was examined in C57BL/6J mice receiving native κ-carrageenans for six weeks, either at 0.5% in drinking water or at 5% in the diet [93]. Under a high-fat background, drinking-water administration increased disease activity, colonic myeloperoxidase activity, and TLR4 mRNA expression, whereas dietary incorporation produced no visible inflammatory effects. Neither condition produced signs of colitis under the low-fat background. Because the regimens differed in both matrix and concentration, the comparison does not isolate the effect of the food matrix or demonstrate that dietary incorporation is inherently protective.

Consistent with this context dependence, carrageenans enhanced inflammatory responses in LPS-related cellular experiments and a *Citrobacter freundii* mouse model [94]. Epithelial–immune-cell interactions have also been implicated in disruption of Caco-2 monolayers [95]. Together, these findings suggest that inflammatory challenge and communication between epithelial and immune cells can influence the intestinal response, rather than carrageenan acting through a single uniform pathway.

Chemically induced colitis models provide further evidence of susceptibility-dependent effects. Carrageenans aggravated trinitrobenzene sulfonic acid (TNBS)-induced inflammation, with changes in cytokines, oxidative stress, and inflammatory signaling [96]. In BALB/c mice, gavage administration of carrageenan in saline at 1.7, 8.3, or 41.7 mg/kg for two weeks before oxazolone challenge aggravated colonic injury and was accompanied by cytokine and TLR4/NF-κB-related changes [97]. Conversely, carrageenan alone did not produce comparable macroscopic or histological damage in that experiment. Changes in regulatory T-cell markers provide additional context but are not, by themselves, evidence of altered innate effector function.

Degraded preparations require separate consideration. A study comparing a carrageenan polymer, mildly degraded material, and oligosaccharides examined microbial degradation and inflammatory effects in germ-free and bacteria-associated mouse experiments [98]. These findings support interactions between molecular form and microbial capacity but do not demonstrate that customary consumption of native food-grade carrageenan invariably generates the same inflammatory products.

Conversely, chemically modified preparations can produce different responses. Acetylated carrageenan preparations reduced TNF-α, IL-1β, and the adhesion molecule ICAM-1 and increased tight-junction protein expression in a Caco-2 model [99]. Such results illustrate structure-dependent epithelial responses; they do not establish equivalent effects of unmodified food-grade carrageenans or protection in humans.

### 6.4. Mechanistic Interpretation

The most extensively investigated inflammatory mechanisms involve TLR4-associated signaling, BCL10, NF-κB, and oxidative pathways. In human intestinal epithelial cells, experimental evidence implicated TLR4 in carrageenan-induced activation of the BCL10–NF-κB–IL-8 pathway [19]. Related work distinguished a BCL10-associated pathway from ROS- and Hsp27-associated signaling [100]. These findings provide a mechanistic basis for epithelial inflammatory responses, although they should not be assumed to explain every response across carrageenan preparations and immune-cell populations.

Structural experiments further demonstrate why degradation cannot be interpreted solely through molecular weight. Enzymatic cleavage of particular galactosidic bonds either reduced or enhanced IL-8 and BCL10 responses, whereas the ROS-associated response was unaffected by the tested enzyme treatments [17]. Thus, the biological consequences of degradation depend on which structural features are removed or exposed. This study supports a role for galactosidic bonds; it does not establish a general relationship between lower molecular weight, increased fermentability, and aggravated colitis.

Carrageenan also enhanced TNF-α-associated inflammatory signaling while inhibiting apoptosis in an epithelial-cell study [101]. This finding illustrates that inflammatory signaling and cell survival can change in different directions. However, it does not directly establish interactions with gut-associated lymphoid tissue or a causal contribution to human inflammatory bowel disease.

Studies of oligosaccharides reveal additional, preparation-dependent mechanisms. In LPS-stimulated BV-2 microglia, κ- and ι-carrageenan acid-hydrolysate oligosaccharide fractions at 125–500 µg/mL reduced TNF-α secretion [102]. TNF-α was more responsive than IL-6 within that experimental comparison, but this does not establish its universal superiority as a carrageenan-related biomarker.

Another microglial study reported reductions in NO, TNF-α, and IL-10 following carrageenan oligosaccharide exposure, with weaker effects after desulfation [103]. However, the accompanying reduction in the viability-associated assay complicates interpretation: lower mediator output may partly reflect fewer or less metabolically active cells. Consequently, these results should not be described as unequivocal protection against inflammation.

More recent work implicated AMPK/ULK1-associated autophagy in the regulation of microglial responses by carrageenan oligosaccharides [104]. This provides a mechanistic lead for the tested derivatives, while therapeutic efficacy and relevance to dietary exposure remain separate questions. Likewise, antioxidant activity reported for degraded carrageenan preparations in chemical assays [105,106] supplies physicochemical context but does not demonstrate improved immune-cell function or host defense.

### 6.5. Evidence Limitations and Relevance to Human Dietary Exposure

The evidence supports preparation- and context-dependent immune modulation rather than a single carrageenan effect. Importantly, pro-inflammatory findings cannot all be attributed to degraded material: native carrageenans have also produced intestinal changes under particular dietary, microbial, or inflammatory conditions [18,93,97]. Conversely, responses obtained with oligosaccharides, acetylated derivatives, or injected preparations cannot establish the effects of ordinary food-grade carrageenan consumption [84].

Evidence without detected adverse effects should therefore be included alongside positive inflammatory findings. In a 90-day dietary study, Fischer 344 rats received κ-carrageenan with a reported molecular-weight range of 196–257 kDa at dietary concentrations of 25,000 or 50,000 ppm. No treatment-related adverse effects were identified in the assessed endpoints [107]. This provides evidence concerning the tested material and feeding conditions, although a general toxicology study does not exclude changes in specialized innate immune functions that it did not measure.

Human evidence remains limited. A National Health and Nutrition Examination Survey (NHANES) analysis examined carrageenan-containing protein supplement beverages and C-reactive protein (CRP), using beverage form as an exposure proxy in its primary analysis [108]. It found no overall association with higher CRP, although subgroup findings suggested possible differences among participants reporting poorer health. These observations are hypothesis-generating: they neither establish total carrageenan intake nor directly assess mucosal immunity, phagocyte function, or causal effects.

Accordingly, concerns about susceptible individuals and inflammatory bowel conditions remain relevant research questions [109], but aggravation of experimental colitis should not be presented as proof that carrageenan initiates human disease. A more informative synthesis requires study-level reporting of subtype, molecular-weight distribution, degradation or modification, purity, dose, vehicle, exposure duration, dietary background, and host condition. Functional immune endpoints should then be evaluated separately from cytokine changes, epithelial injury, and microbiota associations. This approach preserves mechanistic findings while making their implications for human dietary exposure explicit.

## 7. MSG and Innate Immune Responses

### 7.1. Dietary Exposure and Characteristics Relevant to Interpretation

Monosodium glutamate (MSG, E621) is a widely used flavor enhancer that contributes to umami taste and improves the palatability of soups, sauces, processed meats, and other foods [110,111,112,113]. Its biological effects are associated with the glutamate component; nevertheless, added MSG, naturally occurring free glutamate, and protein-bound glutamate should be distinguished when estimating exposure and interpreting experimental findings. EFSA established a group ADI of 30 mg/kg body weight/day, expressed as glutamic acid, for glutamic acid and glutamates E620–E625 used as food additives [114]. This value is distinct from maximum permitted concentrations in particular food categories and from glutamate concentrations measured in biological compartments. Accordingly, an experimental MSG dose should not be compared numerically with the ADI without considering the substance and units in which exposure is expressed.

Exposure assessments indicate variation in glutamate intake across populations and age groups, with children potentially experiencing higher exposure relative to body weight [115,116]. Consequently, a single estimate of “typical human MSG intake” cannot adequately represent all dietary settings. These differences also complicate comparisons between regulatory exposure assessments and experimental studies, whose doses, administration schedules, and food matrices vary substantially [117].

The importance of the food matrix is supported by a rat toxicokinetic study in which concomitant food intake reduced the peak plasma glutamic acid concentration and overall systemic exposure after oral MSG administration, while delaying the time to peak concentration [118]. Thus, gavage or another oral bolus administered without food is not equivalent to MSG consumed with a meal, even when the nominal administered dose is identical.

### 7.2. Effects on Innate Immune Cells and Effector Functions

A 90-day study examined adult male Wistar rats receiving MSG at 20 mg/kg body weight/day by intragastric gavage [20]. Reported findings included leukopenia, changes in differential leukocyte proportions, and alterations in lysozyme and NO-related parameters. The authors reported no change in an endpoint labeled “phagocytic activity”; however, this was assessed by colorimetric NBT reduction rather than direct particle uptake. Moreover, the tested cells were described as neutrophils in Methods and macrophages in Results. This finding therefore cannot establish that phagocytosis was unaffected. Reduced lymphocyte proliferation and immunoglobulin changes primarily concern adaptive immunity. Overall, the study indicates modulation of several immune parameters under the tested conditions, while direct evidence of altered innate effector function remains limited. The single-dose design does not establish a dose–response relationship or an effect threshold, but the administered dose shows that the MSG evidence base is not exclusively high-dose.

Other experiments have predominantly assessed immune-organ injury rather than isolated innate effector functions. In adult male rats, MSG administered by gavage at 3 g/kg body weight/day for eight weeks was associated with oxidative stress, altered IL-1β and IL-10, and disruption of thymic and splenic architecture [119]. Some changes improved after exposure ceased, although recovery differed between tissues. These findings concern a repeated high-dose bolus and include substantial adaptive-immune involvement; they do not directly demonstrate impaired microbial killing or resistance to infection.

Similarly, a rat study using oral MSG at 600 mg/kg body weight/day, alone or with a high-lipid diet, reported splenic abnormalities [120]. This is an in vivo study and should not be presented as evidence from direct incubation of immune cells. Its findings instead contribute to the assessment of immune-organ responses under a defined dietary and metabolic context.

Taken together, the available studies provide evidence of altered immune-cell profiles and immune-tissue responses, but functional innate endpoints remain comparatively limited. In particular, tissue ROS measurements, leukocyte counts, and histological injury should be distinguished from cell-specific oxidative burst, phagocytosis, chemotaxis, and microbial clearance.

### 7.3. Innate Immune Mediators and Barrier–Microbiota Interactions

MSG-associated cytokine changes vary with dose, tissue, and experimental context. In the 90-day rat study, the cytokine panel included mediators associated with both inflammatory and regulatory responses [20]. Accordingly, these findings should not be summarized as a uniform increase in “pro-inflammatory cytokines,” particularly when IL-10 is included. Furthermore, cytokines measured in whole tissues cannot identify their cellular source without additional analyses.

Intestinal studies provide a complementary perspective. In mice, MSG at 30 mg/kg did not significantly alter the assessed serum CRP, intestinal IL-1β, IL-6, TNF-α, secretory IgA, or fecal albumin, whereas the 1500 mg/kg condition was associated with intestinal structural and microbiota disturbances [121]. These findings support dose-dependent differences within the tested model. Nevertheless, neither changes in cardiovascular-associated biomarkers nor shifts in particular bacterial genera establish clinical cardiovascular disease or a universally harmful microbial community. Likewise, the absence of changes in the measured endpoints at the lower dose does not establish a general threshold for all immune effects.

Conversely, a small human study examining MSG consumption at 2 g/day for four weeks reported limited changes in gut microbiota composition and functional features [122]. This is relevant evidence under the tested intake conditions, but it addresses microbiome outcomes rather than directly measuring innate immune-cell function. Its findings therefore should not be extended to exclude all immune effects or responses at other doses and durations.

More direct barrier evidence comes from experiments using Caco-2 cells and gut-on-a-chip models [123]. Exposure to 1% MSG reduced epithelial electrical resistance, increased paracellular flux, and altered occludin staining. ROS increased after 24 h, while transcriptomic findings implicated cellular stress, endoplasmic reticulum stress, mitochondrial processes, and autophagy [123]. These observations support epithelial barrier injury under direct exposure conditions, although cytotoxicity complicates attribution to a specific immune mechanism. They also do not demonstrate downstream dysbiosis or inflammation in an intact host.

A high-fat-diet mouse study additionally linked MSG-associated metabolic and immune alterations with microbiota and short-chain fatty acid changes [124]. Such findings support investigating interactions between exposure and metabolic status. However, microbiota associations and improvement following an intervention do not, by themselves, establish that microbial changes caused the immune phenotype.

### 7.4. Mechanistic Interpretation

Oxidative imbalance and inflammatory signaling recur across MSG experiments, but their immunological interpretation requires attention to the model. A rat study using MSG at 600 mg/kg body weight/day by gavage for 28 days implicated TLR4-associated signaling in thymic and splenic responses and reported attenuation following quercetin treatment [21]. This supports an interaction between redox disturbance and inflammatory pathways under the tested conditions. Nevertheless, improvement with a compound possessing several biological activities does not alone establish that a single pathway is necessary for MSG-associated injury.

In intestinal epithelial experiments, 4 mM N-acetyl-L-cysteine attenuated the ROS increase associated with 1% MSG [123]. This strengthens evidence for a redox component of the cellular response, but does not establish restoration of every barrier endpoint or prevention of immune dysfunction. More generally, ROS accumulation should not be equated with the regulated oxidative burst used by phagocytes during antimicrobial defense.

Neurotoxicity studies provide additional mechanistic context because glutamate participates in neuronal signaling and excessive exposure can engage excitotoxic pathways [125,126]. MSG-related hippocampal changes in neonatal rats and neurotoxicity experiments in adult male rats have implicated mitogen-activated protein kinase (MAPK)- and NF-κB-associated responses [127,128]. Likewise, inflammatory and oxidative changes have been reported in aged-rat spinal cord, testicular, brain, and renal models [129,130,131,132]. These findings demonstrate broader biological responses, but they do not independently establish direct effects on innate immune cells.

Although neuroinflammation can involve innate immune mechanisms [133,134], neuronal injury should therefore remain contextual unless the study identifies a relevant immune-cell response or function. Maternal-exposure findings involving hypothalamic NF-κB and Mammalian Target of Rapamycin (mTOR) signaling in offspring similarly inform developmental and metabolic susceptibility rather than direct dietary immunotoxicity in adults [135]. This distinction prevents the extensive organ-toxicity literature from displacing the central question of innate immune modulation.

### 7.5. Evidence Limitations and Relevance to Human Dietary Exposure

The MSG literature contains heterogeneous exposure conditions and endpoints. Some studies report immune-associated changes at comparatively low administered doses, whereas others employ hundreds or thousands of milligrams per kilogram body weight, repeated bolus administration, or metabolically altered animals [20,21,119,120,121]. Consequently, neither a universal high-dose explanation nor a single dose threshold adequately describes the evidence.

Human studies also require separation by the outcomes they measure. The NutriNet-Santé findings cited here were published as a conference abstract and reported associations between estimated dietary glutamate exposures and cardiovascular outcomes [136]. They provide preliminary epidemiological context, but do not directly assess innate immune function or demonstrate an immune mechanism underlying the association.

Similarly, studies and reviews of MSG-associated symptoms or allergic responses address questions distinct from chronic innate immunotoxicity [117,137]. The reproducibility of symptoms during controlled challenges cannot establish whether long-term exposure alters phagocyte function, just as occasional symptoms cannot demonstrate immune injury. An absolute dose expressed in grams for a human challenge must also be distinguished from an animal dose expressed in grams per kilogram body weight.

Mechanistic support and dietary-exposure relevance should therefore be assessed separately. Pathway changes in exposed tissues or cultured cells establish biological responses, while functional assays determine whether those changes affect immune performance. Toxicokinetic evidence further demonstrates that concomitant food intake can substantially modify internal exposure [118]. Accordingly, translation requires consideration of dose, administration vehicle, food matrix, duration, developmental stage, and metabolic status.

The current evidence supports context-dependent modulation of immune-associated parameters, but remains insufficient to define a consistent adverse innate immune effect at customary human dietary intake. Future studies should combine realistic repeated dietary exposure with cell-specific functional measurements and, where appropriate, host-defense outcomes. The comparative synthesis presented in the following section should maintain this distinction between mechanistic evidence and relevance to consumer exposure. 

## 8. Cross-Compound Synthesis: Shared Mechanisms, Functional Evidence, and Dietary-Exposure Relevance

Although imazalil, TBHQ, carrageenans, and MSG differ in chemical structure and their roles in food-associated exposure, their reported biological effects intersect with redox signaling, cytokine regulation, intestinal barrier–microbiota interactions, and innate immune-cell responses [1,10]. However, convergence on these processes does not establish a common initiating mechanism or equivalent immunotoxic potential. The comparison therefore distinguishes evidence of a molecular or cellular response from evidence of impaired immune function and, separately, from evidence relevant to human dietary exposure.

### 8.1. Redox-Sensitive Signaling and Cytokine Regulation

Redox regulation provides a useful framework for comparison because ROS participate in both antimicrobial defense and cellular signaling, whereas excessive or sustained production can contribute to injury. Conversely, antioxidant responses can limit damage while also modifying inflammatory activation [31,32,33,138]. Thus, neither increased ROS nor enhanced antioxidant signaling has an inherently adverse or protective immunological meaning; interpretation depends on the cells involved and the resulting functional consequences.

TBHQ differs from the other compounds through its well-characterized activity as an Nrf2 activator [16,33,63,67,68]. In macrophage experiments, antioxidant-related transcriptional responses occurred alongside changes in inflammatory mediators and phagocytosis, whereas murine NK-cell experiments showed reduced activation markers and effector-associated proteins [15,60]. Nevertheless, the established ability of TBHQ to activate Nrf2 should not be confused with experimental demonstration that every observed immune effect requires this pathway. The NK-cell and macrophage studies support TBHQ-associated modulation, but do not independently establish Nrf2 dependence for all measured endpoints.

Imazalil, carrageenan preparations, and MSG have also been associated with redox disturbance and inflammatory signaling, although the evidence involves different tissues and exposure conditions [11,12,13,17,21,100,123]. These observations support shared downstream processes rather than an interchangeable mechanism across compounds. In particular, tissue oxidative stress should be distinguished from the regulated oxidative burst of a phagocyte, and altered NF-κB expression or activity should be distinguished from demonstrated failure of host defense.

Cytokine responses are similarly heterogeneous. Changes in inflammatory, regulatory, and tissue-associated mediators may reflect immune activation, compensatory responses, or secondary effects of tissue injury [11,12,13,15,16,17,18,20]. Consequently, an increase or decrease in an individual cytokine cannot be assigned a uniform direction of immunotoxicity. Interpretation requires information about the stimulation context, cellular source, timing, and accompanying functional or tissue-level outcomes.

### 8.2. Intestinal Barrier–Microbiota Interactions

The intestinal interface is relevant to dietary exposure, but the strength and nature of evidence differ between compounds. Imazalil studies have reported microbiota changes, altered mucus or barrier-associated parameters, and intestinal cytokine responses in mice [11,12,13]. These findings support a relationship between exposure and intestinal homeostasis; nevertheless, the simultaneous occurrence of microbial and immune changes does not establish the direction of causality or identify a single responsible pathway.

For carrageenans, experimental evidence more directly illustrates the contribution of microbial and dietary context. A mouse study linked microbiota and mucus-layer changes with increased susceptibility to subsequent infectious inflammation [18]. Another study showed different outcomes when native κ-carrageenan was administered in drinking water or incorporated into the diet, with inflammatory changes depending additionally on a high-fat versus low-fat background [93]. These findings make vehicle and dietary matrix integral parts of interpretation, rather than minor methodological details.

At the same time, epithelial responses cannot be generalized across carrageenan preparations. Studies have reported both an absence of selected permeability or cytokine effects under particular conditions and cytotoxic or barrier-associated changes in other experimental systems [139,140]. The latter study compared κ-, ι-, and λ-carrageenan and found subtype-dependent responses. Accordingly, comparisons require material characterization, concentrations, exposure duration, and viability assessment; neither study establishes the effects of all food-grade preparations during customary consumption.

MSG studies likewise show that intestinal outcomes depend on exposure conditions. Mouse experiments reported different responses at 30 and 1500 mg/kg, while a small human study found limited microbiota modulation during four weeks of intake at 2 g/day [121,122]. Conversely, direct epithelial exposure produced barrier and cellular-stress changes in vitro [123]. These results address different questions: a microbiome study does not directly measure innate immune function, and epithelial injury in culture does not establish an equivalent effect after ingestion.

For TBHQ, the studies emphasized here provide a clearer basis for immune-cell and redox effects than for a shared microbiota-mediated mechanism [15,60]. Consequently, the gut–immune interface is an important axis of comparison, but it should not be presented as an equally demonstrated mechanism for all four compounds.

### 8.3. Innate Immune-Cell Function and Adverse Outcomes

Functional measurements provide information that cytokine profiles and pathway analyses cannot supply alone. However, the reviewed assays vary in what they establish. Human ex vivo neutrophil experiments with imazalil measured particle uptake and chemotaxis, providing direct evidence of altered effector processes under the tested conditions [14]. Neither endpoint directly demonstrates microbial killing or recruitment during infection.

Carrageenan studies include measurements of macrophage motility and phagocytic activity, with responses differing between native polymers, degradation products, and experimental settings [86]. Nevertheless, neutral-red uptake in a macrophage cell-line study should not be treated as equivalent to direct particle engulfment or antimicrobial clearance [85]. Such distinctions are essential when comparing apparently similar “functional” findings across compounds.

TBHQ experiments demonstrate endpoint-specific responses. Macrophage phagocytosis increased at a tested low concentration, while NO and IL-10 responses decreased at a higher non-cytotoxic concentration [60]. In NK cells, reduced IFN-γ, granzyme B, perforin, and activation markers indicate changes in activation and effector potential [15]. However, these measurements do not replace a target-cell killing assay. Protective outcomes reported in a pharmacological antiviral model further show that TBHQ-associated modulation cannot be assigned a uniformly suppressive direction [65].

For MSG, evidence is more heavily weighted toward leukocyte profiles, mediators, and lymphoid-organ responses [20,21,119,120]. The study reported no change in an endpoint labeled phagocytic activity, although this was assessed by colorimetric NBT reduction rather than direct particle uptake; cell identity was reported inconsistently between Methods and Results [20]. Consequently, the reviewed findings indicate immune-related responses, while direct evidence of impaired phagocyte function remains limited.

Together, these studies support context-dependent immune modulation. Adverse interpretation is stronger when cellular changes are linked to impaired clearance, altered infection outcomes, or relevant tissue injury. Nevertheless, experimental adversity and consumer risk remain separate questions: an adverse effect in a defined model does not establish that ordinary dietary exposure produces the same outcome.

### 8.4. Weight of Evidence and Relevance to Dietary Exposure

A meaningful comparison requires separate consideration of mechanistic support and exposure relevance. Mechanistic support depends on the specificity of the measurements, experimental testing of pathway involvement, functional outcomes, consistency across models, and independent replication. Conversely, dietary-exposure relevance depends on the tested material, dose, administration conditions, food matrix, duration, and the relationship between administered and internal exposure.

These dimensions do not necessarily increase together. Human-derived cells may provide useful mechanistic evidence without reproducing human ingestion, while an animal feeding study may approximate dietary administration but measure few specialized immune functions. Likewise, a non-cytotoxic culture concentration is not automatically physiologically relevant, and correspondence with an ADI does not establish that an experimental exposure reproduces consumer intake.

The distinction is particularly important for carrageenan, where native polymers, degraded preparations, oligosaccharides, and poligeenan cannot be combined as a single exposure [84]. Food co-ingestion can also modify systemic glutamate exposure after MSG administration, illustrating why an oral bolus and consumption with meals should be assessed separately [118]. For TBHQ, nominal culture concentrations cannot be converted directly into dietary intake without toxicokinetic information.

Accordingly, Table 1 provides a descriptive comparison rather than a ranking of immunotoxic potency or consumer risk. TBHQ has a well-characterized redox target, but pathway dependence and functional adversity remain endpoint-specific. Imazalil has a focused evidence base involving neutrophil responses and intestinal homeostasis. Carrageenan has substantial structural and experimental heterogeneity, whereas MSG findings vary with dose, administration conditions, and metabolic context. None of these evidence patterns, on its own, establishes a complete chain from customary human dietary exposure to clinically meaningful impairment of innate immunity.

**Table 1:** Cross-compound comparison of reported innate immune-related responses, mechanistic support, and relevance to human dietary exposure.

Compound	Principal Evidence	Mechanistic Interpretation	Dietary Relevance and Key Limitation
Imazalil [11,12,13,14]	Neutrophil uptake/migration and intestinal inflammatory/barrier-associated changes.	Direct neutrophil changes; intestinal causal links remain incomplete.	Human cells do not reproduce ingestion; mouse oral regimens vary. No demonstrated consumer host-defense impairment.
TBHQ [10,15,60,61,62,63]	Macrophage uptake/mediators, NK activation/effector proteins, preliminary influenza observations.	Nrf2 dependence supported for specific models; not all endpoints. Pharmacological antiviral responses can differ in direction.	Cell concentrations, dietary experiments and intraperitoneal treatment are distinct exposure scenarios.
Carrageenans [17,18,19,85,86,87,93,107,108,139,140]	Preparation-dependent phagocyte and epithelial responses; vehicle/challenge-dependent intestinal findings; relevant null results.	Structural, epithelial-signaling and microbiota evidence must remain model-specific.	Subtype, molecular weight, modification, matrix and host condition preclude a single general effect.
MSG [20,21,119,120,121,122,123]	Immune profiles/organ changes; unchanged author-labeled NBT-based “phagocytic activity” in a key study; intestinal and human microbiome observations.	Tissue redox/signaling changes are not equivalent to innate-cell dysfunction.	Dose, bolus delivery, metabolic context and matrix matter. Human microbiome data do not measure innate function.

Mechanistic support and human dietary-exposure relevance are assessed separately. The descriptions summarize the nature and consistency of the reviewed evidence; they are not formal risk-of-bias grades, rankings of immunotoxic potency, or estimates of consumer risk. Study-level details are provided in Tables S1–S4. E407 designates carrageenan as a food additive and is not specific to the κ subtype.

## 9. Limitations and Evidence Gaps

The evidence reviewed for imazalil, TBHQ, carrageenans, and MSG originates predominantly from animal experiments, cell cultures, and short-term ex vivo studies, with limited confirmation in humans. These approaches provide complementary information, but substantial differences in exposure conditions, biological systems, and measured endpoints restrict direct comparison. Moreover, several experiments investigated general toxicity, metabolic disturbance, or tissue inflammation rather than innate immunotoxicity as a primary outcome. Consequently, immune-associated findings must be evaluated in relation to the original study design: a change detected during organ injury may have a different interpretation from a response demonstrated in viable immune cells or an exposure-associated alteration in resistance to infection [3,24].

These limitations do not invalidate experimental evidence. Rather, they determine which conclusions a study can support and where further confirmation is required. Molecular responses, cellular functions, tissue injury, and organism-level outcomes represent different levels of evidence, while relevance to dietary exposure constitutes a separate dimension. Their integration is therefore essential for distinguishing a biologically plausible immune effect from an adverse response demonstrated under experimental conditions and, ultimately, from a risk relevant to consumers.

### 9.1. Model Relevance and Characterization of Exposure

Animal models capture interactions among metabolism, epithelial barriers, intestinal microbiota, and systemic immunity that cannot be reproduced in isolated cells. Nevertheless, species, strain, sex, age, and metabolic status can influence both internal exposure and immune responsiveness. Translation therefore requires consideration of toxicokinetic and biological differences rather than reliance on administered dose alone [141,142]. Fish models provide additional opportunities to examine intestinal inflammation and host defense, but their immune organization, physiology, and exposure conditions limit direct extrapolation to mammalian dietary responses [143,144].

The reviewed evidence is not uniformly restricted to high doses or non-dietary administration. However, its exposure conditions vary sufficiently that a general classification as either “dietary-relevant” or “supraphysiological” would obscure important differences. MSG studies, for example, include comparatively lower-dose oral experiments alongside repeated high-dose bolus administration and models involving altered metabolic conditions [20,119,121,124]. Likewise, native carrageenans have produced different intestinal outcomes depending on the administration vehicle and dietary background [93]. Exposure relevance should consequently be evaluated study by study rather than inferred from the compound name or the oral route alone.

Administered dose must also be distinguished from internal exposure. A dose expressed in mg/kg body weight does not specify the concentration reaching an immune tissue, its duration, or the contribution of metabolites. Similarly, a dietary concentration does not define the daily ingested dose without information about food consumption and body weight. Repeated gavage, drinking-water administration, and incorporation into food may differ in exposure timing and peak concentrations, even when estimated daily doses are similar. Comparisons with human intake or regulatory reference values therefore require compatible units and a justified toxicokinetic basis; numerical proximity to an ADI is not sufficient to establish equivalent exposure.

For carrageenan, these considerations are compounded by material heterogeneity. Subtype, molecular-weight distribution, degradation method, sulfate content, purity, and formulation can influence biological responses. Food-grade polymers, deliberately degraded preparations, oligosaccharides, and poligeenan must therefore remain distinguishable [56,84]. Nevertheless, molecular weight alone cannot account for all discrepancies: the food matrix, vehicle, host microbiota, and inflammatory state also influence the experimental outcome [18,93]. Where characterization is incomplete, uncertainty should be retained explicitly rather than resolved by assuming that a preparation was either native or degraded.

In vitro and ex vivo studies introduce a different exposure problem because compounds are applied directly to cells without the integrated processes of absorption, first-pass metabolism, distribution, and elimination. Nominal concentrations in culture medium may differ from freely available concentrations and from those achievable in intestinal or systemic compartments. Compound stability, binding to medium components, and exposure duration can further affect the cellular dose. Accordingly, extrapolation requires toxicokinetic information and identification of the biological compartment represented by the assay [145,146,147,148]. Repeated exposure may also produce adaptation or delayed effects that a single short incubation does not capture; conversely, prolonged direct contact in culture may not reproduce the changing concentrations experienced in vivo.

### 9.2. Cellular Models, Assay Specificity, and Adversity

Cellular systems permit controlled examination of signaling and effector processes, but their responses depend on cell origin, differentiation state, culture conditions, and stimulation protocol [149,150]. Immortalized lines offer reproducibility and experimental accessibility, yet may differ from primary cells in phenotype and inflammatory responsiveness, as discussed for RAW 264.7 macrophages [151]. Primary cells improve biological relevance but introduce donor variability and can undergo changes during isolation and culture. Neither approach is therefore intrinsically sufficient: confidence increases when findings are reproduced in complementary systems that address the same biological question.

Human ex vivo neutrophil experiments improve species relevance, but direct incubation remains distinct from dietary exposure [14]. Their interpretation also depends on the cellular preparation. Responses measured in purified or enriched cells may differ from those obtained in mixed leukocyte preparations, where interactions among cell populations can contribute to the outcome. Likewise, effects observed in unstimulated cells should be distinguished from those detected after LPS, receptor agonists, or pharmacological activation. Suppression of an experimentally induced response does not necessarily indicate impaired basal function, while enhancement of a resting-cell signal does not necessarily improve performance during infection.

Assay specificity is particularly important when interpreting functional evidence. Particle uptake establishes an engulfment-related process but does not measure intracellular killing or pathogen clearance. Chemotaxis in an experimental chamber measures migration under defined conditions rather than successful recruitment within an infected tissue [14]. Similarly, NK-cell activation markers and granzyme or perforin expression indicate activation and effector potential, but do not replace a target-cell killing assay [15]. Neutral-red uptake should also be distinguished from direct particle-engulfment measurements [85]. These endpoints remain informative, provided that the conclusions reflect what was actually measured.

Comparable caution applies to oxidative endpoints. ROS accumulation in a tissue homogenate or epithelial culture does not identify its cellular source and should not be equated with a regulated phagocyte oxidative burst. Extracellular DNA release can accompany several cellular processes and therefore requires complementary evidence before being attributed to NET formation. Differences in assay timing are also consequential: an early activation response, a delayed loss of function, and a recovery-phase measurement may describe different stages of the same biological process rather than contradictory effects.

Viability assessment is necessary because reduced mediator output or apparent suppression of activation can accompany cell loss or altered metabolic activity [103]. However, a viability-associated metabolic assay does not independently identify the mechanism of cell injury, and preservation of viability does not establish preservation of immune competence. Consequently, immune endpoints should be interpreted alongside appropriate measures of cell number and viability, while recognizing that an effect at a non-cytotoxic concentration is not automatically adverse or exposure-relevant.

Cytokine, transcriptional, and oxidative-stress findings can contribute to hazard identification, but an isolated change does not establish an adverse immune effect [3]. Their significance is stronger when supported by convergent functional evidence, relevant tissue injury, or organism-level outcomes [24,25]. Conversely, unchanged phagocytosis does not exclude effects on other immune functions, just as altered cytokines do not demonstrate that phagocytosis or microbial killing has been impaired. A balanced interpretation must therefore retain both positive and unchanged endpoints rather than selecting only those consistent with a general label of immune stimulation or suppression [20,60].

### 9.3. Causality, Replication, and Evidence Coverage

A recurring limitation is the distance between pathway-associated observations and causal demonstration. Changes in receptor expression, transcription-factor activity, or pathway-enriched gene sets identify candidate mechanisms, but do not establish that a pathway is necessary for the measured response. Confidence is greater when pathway involvement is tested experimentally, and the intervention changes the relevant functional outcome. This distinction is particularly important when a compound has a well-established molecular activity, such as Nrf2 activation by TBHQ: that activity cannot automatically be assigned as the cause of every immune endpoint observed after exposure [15,60].

Experiments using protective interventions require similar caution. Attenuation of an MSG-associated response by quercetin supports the biological responsiveness of the model, but a compound with multiple antioxidant and signaling activities does not isolate a single causal mechanism [21]. Moreover, improvement in biochemical or histological markers does not necessarily establish restoration of host defense. Protective-intervention studies are therefore useful mechanistic evidence, provided that prevention of a particular experimental phenotype is distinguished from confirmation of a complete exposure-to-adversity pathway.

For microbiota-associated effects, the central question is whether microbial changes initiate an immune response, arise from tissue injury, or occur in parallel with other exposure effects. This uncertainty is not uniform across studies. Carrageenan experiments incorporating fecal transfer provide evidence of a microbiota-mediated contribution under specific conditions [18]. Nevertheless, transfer of an exposure-associated phenotype does not by itself identify the responsible organism, metabolite, or microbial interaction, nor establish that the mechanism operates with another preparation or in humans. Broader interpretation remains constrained by variation in donor communities, recipient conditions, dietary background, and experimental design [152].

Independent replication is also uneven. Several influential imazalil findings concerning intestinal homeostasis and maternal or multigenerational exposure originate from a limited set of related studies [11,12,13]. These studies contribute distinct observations, but confirmation across laboratories, biological backgrounds, and exposure designs would strengthen generalizability. More broadly, evidence coverage should not be confused with replication: multiple reports of oxidative stress in different organs do not necessarily confirm the same innate immune mechanism, and several endpoints measured in one experiment do not constitute independent evidence.

Dose–response information is similarly incomplete. A single-dose experiment can establish an effect under the tested conditions but cannot define an effect threshold, response shape, or exposure range over which the finding persists. Conversely, several tested doses do not automatically establish a general non-monotonic relationship, particularly when endpoint-specific responses have not been independently reproduced. Exposure duration and recovery measurements introduce further dimensions: reversibility may help characterize persistence, but does not establish that an effect was harmless while present.

Finally, the literature encompasses biological outcomes that are relevant but not interchangeable. Adaptive immunity, tumor responses, neurotoxicity, developmental effects, and findings in non-mammalian species can inform mechanisms or susceptibility. However, they should remain distinguishable from direct evidence of altered innate immune function following dietary exposure. This separation is particularly important in narrative synthesis, where a large body of contextual literature can otherwise create an impression of stronger direct evidence than is available.

### 9.4. Human Evidence and Boundaries of Inference

The principal translational gap is the limited availability of human studies combining characterized dietary exposure with objective innate immune endpoints. Human-derived cell experiments, microbiome studies, symptom challenges, and epidemiological associations each address different questions [14,108,122,136]. Their integration is informative, but one evidence type cannot substitute for another. In particular, absence of a microbiota change does not establish preservation of phagocyte function, and an association with a systemic inflammatory marker does not identify the affected immune-cell population or demonstrate causality.

Human observational studies also depend on the accuracy and scope of exposure assessment. Food categories, ingredient information, or beverage form may serve as exposure proxies without quantifying the total ingested dose [108]. Associations can additionally reflect correlated dietary patterns, metabolic conditions, or other exposures. These limitations do not make observational findings uninformative, but they constrain attribution to an individual compound and require distinction between hypothesis-generating associations and confirmed immune effects.

Clinical relevance further depends on whether a response is local or systemic, transient or persistent, and compensatory or damaging. A short-lived cytokine change may differ substantially from sustained loss of an effector function, while intestinal injury may have immunological significance without producing a measurable systemic inflammatory response. Consequently, failure to detect a systemic biomarker does not exclude a local effect, and a local experimental response does not establish clinically meaningful systemic impairment.

These uncertainties constrain conclusions about consumer risk without negating the experimental findings. The reviewed evidence supports compound- and context-dependent biological responses, but their persistence and adverse significance at customary human dietary exposure remain incompletely established. The following section therefore focuses on research priorities that connect characterized exposure and internal dose with molecular responses, immune function, and relevant adverse outcomes, rather than treating additional biomarker measurements alone as sufficient resolution of the current gaps.

## 10. Implications for Risk Assessment and Research Priorities

### 10.1. Implications for Food-Safety Risk Assessment

The findings reviewed here have implications for the selection and interpretation of immune endpoints in food-safety assessment, although they do not establish adverse effects at customary human dietary exposure. Regulatory context also differs among the compounds: TBHQ, carrageenan, and MSG are evaluated as food additives, whereas imazalil is assessed within pesticide frameworks, including consideration of residues in food [153,154]. Consequently, their comparison is useful for identifying shared biological questions but should not imply identical authorization procedures or exposure limits.

Current food-additive assessments consider established toxicological endpoints and can require additional immunotoxicity testing when available evidence indicates potential immune effects [153]. The central challenge is therefore to determine whether the experimental findings reviewed here warrant more targeted investigation and, if so, which endpoints would be informative. Changes in cytokines, oxidative stress markers, or immune-related transcription may help identify candidate mechanisms, but their contribution to hazard characterization depends on their association with functional changes, tissue injury, or other adverse outcomes [3].

Similarly, the derivation of an ADI from experimental toxicology does not, by itself, demonstrate that chronic low-dose effects have been overlooked. Rather, uncertainty arises where exposure duration, internal dose, or immune endpoints have not been adequately characterized. Although non-monotonic responses have been discussed extensively in other toxicological contexts, particularly endocrine disruption, such observations cannot establish non-monotonic innate immune effects for the four compounds considered here [155,156]. Appropriate dose–response studies are therefore needed before concluding that conventional approaches underestimate their immunological effects.

Microbiota composition, epithelial barrier function, and immune signaling may provide additional information where these processes are implicated in a compound’s activity [157,158,159,160]. Nevertheless, an altered microbial profile is not inherently adverse, and its interpretation requires evidence of associated metabolic, epithelial, or immune consequences. Integrating these measurements with functional outcomes could strengthen assessment without treating every molecular or microbial change as evidence of consumer risk.

### 10.2. Exposure-Relevant Studies and Individual Susceptibility

Resolving these uncertainties requires experimental designs that connect administered exposure with the concentrations and chemical forms encountered at relevant biological sites. Repeated oral administration through a characterized diet is particularly informative for food-associated compounds because the food matrix can influence dissolution, digestion, microbial transformation, and absorption. Drinking-water exposure may also be useful, but it should be distinguished from administration with food. Likewise, gavage offers controlled dosing while producing bolus exposure kinetics that may differ from those associated with distributed dietary intake.

Dose selection should incorporate estimated consumption, relevant regulatory reference values, and compound-specific toxicokinetics. Simple body-surface-area conversion can provide an initial comparison between animal and human doses, but it cannot establish equivalent internal exposure or immunological sensitivity [161]. An ADI should therefore be treated as a regulatory reference rather than an experimental target whose numerical matching proves dietary relevance. Where possible, measurements of the parent compound and relevant metabolites should accompany functional assessments, allowing effects to be related to internal exposure.

For in vitro and ex vivo studies, concentration selection should similarly be informed by available toxicokinetic data or physiologically based kinetic modeling [162]. Nominal medium concentrations may differ from the concentrations available to cells because of protein binding, adsorption, instability, or metabolism [163]. Moreover, concentrations plausible within the intestinal lumen may not be plausible in blood or peripheral immune tissues. These distinctions are especially important when extrapolating effects in isolated immune cells to oral exposure.

Exposure duration and sampling schedules should allow transient responses to be distinguished from persistent changes. Repeated measurements and exposure-free recovery periods can help determine whether an effect resolves, accumulates, or emerges through compensatory processes. Long-term mixture experiments reporting redox adaptations and perturbations illustrate the importance of dose and time, although such findings do not constitute direct evidence of innate immune impairment by the four compounds reviewed here [164].

Individual susceptibility should also be examined rather than assumed. Childhood dietary exposure and additive-associated hypersensitivity provide reasons to consider life stage and pre-existing conditions, but they do not establish greater innate immune sensitivity to all four compounds [165,166]. Studies should therefore evaluate sex, age, metabolic status, baseline inflammation, and relevant disease states while distinguishing responses in otherwise healthy organisms from effects that depend on an existing inflammatory or metabolic disorder. Human investigations, where feasible, could combine dietary exposure assessment with repeated immune measurements and functional assays, while accounting for background diet, medication, infection, and other major determinants of immune status.

### 10.3. Mixtures and Persistent Innate Immune Responses

Because food-associated exposures occur within complex diets, single-compound studies cannot fully resolve the consequences of co-exposure. However, simultaneous exposure does not necessarily imply synergistic toxicity. Mixture experiments should include individual-compound controls and an explicit reference model against which additive, antagonistic, or synergistic responses can be evaluated [167]. Combinations and relative concentrations should be selected from documented dietary co-exposure rather than solely because the compounds share a proposed mechanism or appear in the same review.

The available mixture evidence also requires balanced interpretation. For example, a study examining eight common food additives, individually and in combination, did not detect significant changes in the assessed short-chain fatty acid production or microbiota dysbiosis parameters [168]. This finding neither establishes universal mixture safety nor supports a general claim of microbiota-mediated synergy. Instead, it demonstrates the importance of specifying the tested mixture, exposure conditions, model, and endpoints. Future investigations should extend such comparisons to epithelial and immune functions where justified, allowing microbial changes—or their absence—to be interpreted alongside host responses.

Whether these compounds induce persistent innate immune reprogramming remains unresolved. Testing trained immunity would require an initial exposure, a recovery period sufficient to exclude residual compound activity, and a subsequent challenge, with functional secondary responses assessed alongside metabolic or epigenetic changes. Persistent injury, altered cell composition, and immune tolerance would need to be distinguished from trained immunity.

### 10.4. Functional Endpoints, Multi-Omics, and Candidate Adverse Outcome Pathways

Across these research priorities, endpoint selection should follow the biological question. Phagocytosis and microbial killing are relevant when phagocyte function is implicated, whereas target-cell killing is needed to establish changes in NK cytotoxicity. Chemotaxis, stimulus-dependent oxidative burst, barrier integrity, and resistance to infection may provide complementary information in appropriate models. No single assay panel is sufficient for every compound or mechanism; instead, functional measurements should test whether the molecular changes identified in a study have meaningful consequences [3].

Primary human immune cells, intestinal organoids, epithelial–immune co-cultures, and microbiota-containing systems can help investigate these relationships under controlled conditions. Their value lies in addressing specific limitations of conventional models rather than replacing all animal evidence. Integrated designs could, for example, test whether an epithelial or microbial perturbation generates signals that subsequently alter immune-cell function, and whether these effects are reversible or dependent on the host’s inflammatory state.

Multi-omics approaches offer additional opportunities to identify coordinated changes in transcription, proteins, metabolites, and microbial activity [169,170]. Nevertheless, molecular signatures remain exploratory unless they are reproduced and linked to relevant biological functions. A shared set of reporting requirements—covering material characterization, exposure conditions, viability, sampling time, and endpoint definitions—would improve comparability, while functional assays could be selected according to the suspected mechanism.

An adverse outcome pathway framework can help organize this evidence by distinguishing molecular initiating events, intermediate key events, and adverse outcomes [3,171]. However, a changed transcript or enrichment of an immune-related pathway should not automatically be designated a molecular initiating event. Candidate relationships require evidence of temporal order, dose concordance, biological plausibility, and, where feasible, experimental perturbation showing that an upstream event contributes to a downstream effect (Table 2).

**Table 2:** Candidate relationships for organizing future mechanistic studies.

Candidate Sequence	Evidence Supporting Parts of the Sequence	Knowledge Gap
Redox signaling → immune-cell activation → effector change → altered infection control	TBHQ cellular and animal studies address different parts of this sequence [15,60,63,65]; influenza abstracts add preliminary observations [61,62].	The entire sequence is not demonstrated in one dietary model. Pathway necessity, internal exposure, and direct functional outcomes have not been established
Intestinal exposure → microbiota/barrier alteration → immune signaling → persistent injury or dysfunction	Imazalil and carrageenan studies examine microbial, mucus, mediator, and challenge-associated outcomes [11,12,13,18,93].	Resolve temporal direction, microbial versus epithelial contributions, material/matrix effects, and persistence remain unresolved. These sequences are hypotheses, not validated adverse outcome pathways (AOPs).

These candidate sequences should guide hypothesis testing rather than imply a complete causal chain for every compound. Quantitative links between exposure, key events, and adverse outcomes would strengthen mechanistic confidence, but risk characterization would still require information on human exposure and susceptibility.

A coordinated strategy could therefore combine exposure-informed mechanistic experiments, repeated oral studies with targeted functional endpoints, and human investigations where justified. Controlled dietary studies within applicable safety constraints could assess reversibility and short-term functional responses, whereas longitudinal studies incorporating exposure biomarkers could examine longer-term associations. Together, these approaches would help determine which experimentally observed changes represent transient or compensatory responses and which, if any, translate into adverse immune outcomes during human dietary exposure.

## 11. Conclusions

This review examined evidence on the effects of four structurally and functionally distinct food-associated xenobiotics—imazalil, TBHQ, carrageenans, and MSG—on cellular and humoral components of innate immunity. Despite their different chemical properties and dietary sources, reported effects involve several interconnected processes, including redox-sensitive signaling, cytokine regulation, intestinal barrier–microbiota interactions, and innate immune cell function. Nevertheless, these shared processes do not imply equivalent mechanisms, adverse effects, or consumer risks across compounds.

TBHQ has a comparatively well-characterized connection to Nrf2 activation, alongside experimental evidence of altered macrophage and NK cell responses; however, Nrf2 dependence has not been established for every reported immune effect. Evidence for imazalil is more limited in breadth and includes altered neutrophil functions and intestinal homeostasis, with insufficient independent replication and confirmation during human dietary exposure. For carrageenans, interpretation requires careful differentiation of food-grade polymers from degraded preparations and other derivatives while also accounting for vehicle, host condition, and experimental model. The MSG literature is broader, but its relevance varies substantially with dose, administration route, life stage, and metabolic context.

Taken together, the findings support context-dependent innate immune modulation in experimental systems. However, the predominantly preclinical evidence does not establish clinically meaningful impairment of innate immunity at customary human dietary exposure. Changes in cytokines, oxidative stress markers, or immune-related gene expression can inform mechanistic interpretation, but do not alone demonstrate adversity. Conversely, altered cellular functions warrant further investigation without necessarily establishing impaired host defense in an intact organism.

The principal unresolved question is therefore whether the observed changes occur at relevant internal exposures, persist beyond exposure, and translate into adverse functional outcomes. Addressing this question requires exposure-informed studies that link molecular findings to appropriate immune functions and organism-level consequences. Such evidence would clarify the contribution of innate immune endpoints to food-safety assessment while maintaining the distinction between mechanistic plausibility, hazard identification, and risk under actual dietary conditions.

## Data Availability

No new data were created or analyzed in this study. Data sharing is not applicable to this article.

## Supplementary Materials

The following supporting information can be downloaded at: https://www.mdpi.com/article/10.3390/ijms27219580/s1.

## Abbreviations

The following abbreviations are used in this manuscript:

ADI	Acceptable Daily Intake
AOP	Adverse Outcome Pathway
CCL2	Chemokine (C-C motif) Ligand 2
CD	Cluster of Differentiation
CGN	Carrageenan
CRP	C-Reactive Protein
DNA	Deoxyribonucleic Acid
EFSA	European Food Safety Authority
ICAM-1	Intercellular Adhesion Molecule 1
IFN-γ	Interferon-γ
Ig	Immunoglobulin
IL	Interleukin
IMZ	Imazalil
JECFA	Joint FAO/WHO Expert Committee on Food Additives
LPS	Lipopolysaccharide
MAPK	Mitogen-Activated Protein Kinase
mRNA	Messenger Ribonucleic Acid
MSG	Monosodium Glutamate
mTOR	Mammalian Target of Rapamycin
NBT	Nitroblue Tetrazolium
NF-κB	Nuclear Factor kappa B
NHANES	National Health and Nutrition Examination Survey
NK	Natural Killer
NO	Nitric Oxide
Nrf2	Nuclear Factor Erythroid 2-Related Factor 2
RNA	Ribonucleic Acid
ROS	Reactive Oxygen Species
TBHQ	tert-Butylhydroquinone
Th	T Helper Cell
TLR	Toll-Like Receptor
TNF-α	Tumor Necrosis Factor-α
TNBS	Trinitrobenzene Sulfonic Acid
κ-CGN	κ-Carrageenan

## References

[1] Paula Neto H.A., Ausina P., Gomez L.S., Leandro J.G.B., Zancan P., Sola-Penna M. (2017). Effects of Food Additives on Immune Cells As Contributors to Body Weight Gain and Immune-Mediated Metabolic Dysregulation. Front. Immunol..

[2] Wang H., Bai J., Miao P., Wei Y., Chen X., Lan H., Qing Y., Zhao M., Li Y., Tang R. (2024). The Key to Intestinal Health: A Review and Perspective on Food Additives. Front. Nutr..

[3] Germolec D.R., Lebrec H., Anderson S.E., Burleson G.R., Cardenas A., Corsini E., Elmore S.E., Kaplan B.L., Lawrence B.P., Lehmann G.M. (2022). Consensus on the Key Characteristics of Immunotoxic Agents as a Basis for Hazard Identification. Environ. Health Perspect..

[4] Urrutia-Pereira M., Fogelbach G.G., Chong-Neto H.J., Solé D. (2025). Food Additives and Their Impact on Human Health. Allergol. ET Immunopathol..

[5] Marshall J.S., Warrington R., Watson W., Kim H.L. (2018). An Introduction to Immunology and Immunopathology. Allergy Asthma Clin. Immunol..

[6] Turvey S.E., Broide D.H. (2010). Innate Immunity. J. Allergy Clin. Immunol..

[7] Murphy K., Weaver C., Janeway C. (2017). Janeway’s Immunobiology.

[8] Chandrasekar V., Panicker A.J., Dey A.K., Mohammad S., Chakraborty A., Samal S.K., Dash A., Bhadra J., Suar M., Khare M. (2024). Integrated Approaches for Immunotoxicity Risk Assessment: Challenges and Future Directions. Discov. Toxicol..

[9] International Programme on Chemical Safety (2012). Guidance for Immunotoxicity Risk Assessment for Chemicals.

[10] Seto T., Grondin J.A., Khan W.I. (2025). Food Additives: Emerging Detrimental Roles on Gut Health. FASEB J..

[11] Jin C., Zeng Z., Fu Z., Jin Y. (2016). Oral Imazalil Exposure Induces Gut Microbiota Dysbiosis and Colonic Inflammation in Mice. Chemosphere.

[12] Jin C., Yuan X., Wang C., Fu Z., Jin Y. (2021). Maternal Exposure to Imazalil Disrupts Intestinal Barrier and Bile Acids Enterohepatic Circulation Tightly Related IL-22 Expression in F0, F1 and F2 Generations of Mice. J. Hazard. Mater..

[13] Jin C., Xia J., Wu S., Tu W., Pan Z., Fu Z., Wang Y., Jin Y. (2018). Insights into a Possible Influence on Gut Microbiota and Intestinal Barrier Function during Chronic Exposure of Mice to Imazalil. Toxicol. Sci..

[14] Ratajczak-Wrona W., Aniszewska A., Iwaniuk A., Garley M., Wołczyński S., Skarżynski D.J., Wrobel J., Zebrowska A., Radziwon P., Pucelik B. (2026). “Protective” Imazalil vs Its Negative Effects on Immune System Cells—Neutrophils. Life.

[15] Boss A.P., Freeborn R.A., Duriancik D.M., Kennedy R.C., Gardner E.M., Rockwell C.E. (2018). The Nrf2 Activator tBHQ Inhibits the Activation of Primary Murine Natural Killer Cells. Food Chem. Toxicol..

[16] Turley A.E., Zagorski J.W., Rockwell C.E. (2015). The Nrf2 Activator tBHQ Inhibits T Cell Activation of Primary Human CD4 T Cells. Cytokine.

[17] Bhattacharyya S., Liu H., Zhang Z., Jam M., Dudeja P.K., Michel G., Linhardt R.J., Tobacman J.K. (2010). Carrageenan-Induced Innate Immune Response Is Modified by Enzymes That Hydrolyze Distinct Galactosidic Bonds. J. Nutr. Biochem..

[18] Wu W., Zhou J., Xuan R., Chen J., Han H., Liu J., Niu T., Chen H., Wang F. (2022). Dietary κ-Carrageenan Facilitates Gut Microbiota-Mediated Intestinal Inflammation. Carbohydr. Polym..

[19] Bhattacharyya S., Gill R., Chen M.L., Zhang F., Linhardt R.J., Dudeja P.K., Tobacman J.K. (2008). Toll-like Receptor 4 Mediates Induction of the Bcl10-NFκB-Interleukin-8 Inflammatory Pathway by Carrageenan in Human Intestinal Epithelial Cells. J. Biol. Chem..

[20] Motwadie M.E., Hashem M.M., Abo-EL-Sooud K., Abd-Elhakim Y.M., El-Metwally A.E., Ali H.A. (2021). Modulation of Immune Functions, Inflammatory Response, and Cytokine Production Following Long-Term Oral Exposure to Three Food Additives; Thiabendazole, Monosodium Glutamate, and Brilliant Blue in Rats. Int. Immunopharmacol..

[21] Das D., Banerjee A., Manna K., Sarkar D., Shil A., Sikdar M., Mukherjee S., Maji B.K. (2024). Quercetin Counteracts Monosodium Glutamate to Mitigate Immunosuppression in the Thymus and Spleen via Redox-Guided Cellular Signaling. Phytomedicine.

[22] Auguste M., Melillo D., Corteggio A., Marino R., Canesi L., Pinsino A., Italiani P., Boraschi D. (2022). Methodological Approaches to Assess Innate Immunity and Innate Memory in Marine Invertebrates and Humans. Front. Toxicol..

[23] DeWitt J.C., Germolec D.R., Luebke R.W., Johnson V.J. (2016). Associating Changes in the Immune System with Clinical Diseases for Interpretation in Risk Assessment. CP Toxicol..

[24] Germolec D., Luebke R., Rooney A., Shipkowski K., Vandebriel R., Van Loveren H. (2017). Immunotoxicology: A Brief History, Current Status and Strategies for Future Immunotoxicity Assessment. Curr. Opin. Toxicol..

[25] Ogungbesan A., Neal-Kluever A., Rice P. (2019). Exploring the Use of Current Immunological Assays for the Developmental Immunotoxicity Assessment of Food Contact Materials. Food Chem. Toxicol..

[26] Torfs K., Vermeersch G., Gouwy M., Devos T., Proost P., Struyf S. (2026). Neutrophils as Critical Orchestrators of Chronic Inflammation. Cell Mol. Immunol..

[27] Canton M., Sánchez-Rodríguez R., Spera I., Venegas F.C., Favia M., Viola A., Castegna A. (2021). Reactive Oxygen Species in Macrophages: Sources and Targets. Front. Immunol..

[28] Vivier E., Tomasello E., Baratin M., Walzer T., Ugolini S. (2008). Functions of Natural Killer Cells. Nat. Immunol..

[29] Kandarian F., Sunga G.M., Arango-Saenz D., Rossetti M. (2017). A Flow Cytometry-Based Cytotoxicity Assay for the Assessment of Human NK Cell Activity. JoVE.

[30] Shabrish S., Gupta M., Madkaikar M. (2016). A Modified NK Cell Degranulation Assay Applicable for Routine Evaluation of NK Cell Function. J. Immunol. Res..

[31] Gostner J.M., Becker K., Ueberall F., Fuchs D. (2015). The Good and Bad of Antioxidant Foods: An Immunological Perspective. Food Chem. Toxicol..

[32] Manful C.F., Fordjour E., Subramaniam D., Sey A.A., Abbey L., Thomas R. (2025). Antioxidants and Reactive Oxygen Species: Shaping Human Health and Disease Outcomes. Int. J. Mol. Sci..

[33] Ma Q. (2013). Role of Nrf2 in Oxidative Stress and Toxicity. Annu. Rev. Pharmacol. Toxicol..

[34] Bottazzi B., Doni A., Garlanda C., Mantovani A. (2010). An Integrated View of Humoral Innate Immunity: Pentraxins as a Paradigm. Annu. Rev. Immunol..

[35] Mantovani A., Garlanda C. (2023). Humoral Innate Immunity and Acute-Phase Proteins. N. Engl. J. Med..

[36] Ricklin D., Hajishengallis G., Yang K., Lambris J.D. (2010). Complement: A Key System for Immune Surveillance and Homeostasis. Nat. Immunol..

[37] Marcus A., Raulet D.H. (2013). Evidence for Natural Killer Cell Memory. Curr. Biol..

[38] Wu L., Ye J., Li J. (2026). The Linkage between Innate and Adaptive Immunity: Recent Advances in Antigen Recognition, Processing, and Presentation in Fish. Aquac. Fish..

[39] Vitetta E.S., Fernandez-Botran R., Myers C.D., Sanders V.M., Dixon F.J. (1989). Cellular Interactions in the Humoral Immune Response. Advances in Immunology.

[40] Imazalil (Ref: R 023979). https://aeru.herts.ac.uk/aeru/iupac/Reports/390.htm.

[41] Altieri G., Di Renzo G.C., Lanza G. Imazalil On-Line Control in Post-Harvest Treatments of Citrus Fruit. Proceedings of the V International Postharvest Symposium 682.

[42] Hamrani M., Zelmat L., Jazayeri S.M., El Ammari M., Brhadda N., Ziri R., Aarrouf J., El Guilli M. (2025). Generally Recognized as Safe Salts for a Natural Strategy to Managing Fungicide-Resistant Penicillium Strains in the Moroccan Citrus Packinghouse. Agriculture.

[43] Siegel M.R., Ragsdale N.N. (1978). Antifungal Mode of Action of Imazalil. Pestic. Biochem. Physiol..

[44] Lewis K.A., Tzilivakis J., Warner D.J., Green A. (2016). An International Database for Pesticide Risk Assessments and Management. Hum. Ecol. Risk Assess. Int. J..

[45] Ruiz-Rodríguez L., Aguilar A., Díaz A.N., Sánchez F.G. (2015). Enantioseparation of the Fungicide Imazalil in Orange Juice by Chiral HPLC. Study on Degradation Rates and Extractive/Enrichment Techniques. Food Chem..

[46] Stojanović I., Saksida T., Miljković Đ., Pejnović N. (2021). Modulation of Intestinal ILC3 for the Treatment of Type 1 Diabetes. Front. Immunol..

[47] Fachi J.L., Di Luccia B., Gilfillan S., Chang H.-W., Song C., Cheng J., Cella M., Vinolo M.A., Gordon J.I., Colonna M. (2024). Deficiency of IL-22–Binding Protein Enhances the Ability of the Gut Microbiota to Protect against Enteric Pathogens. Proc. Natl. Acad. Sci. USA.

[48] Guan J., Dewaard M.A. (1993). Inhibition of Sterol 14α-Demethylase Activity in *Penicillium Italicum* Does Not Correlate with Resistance to the DMI Fungicide Imazalil. Pestic. Biochem. Physiol..

[49] Jin C., Zhang R., Fu Z., Jin Y. (2019). Maternal Exposure to Imazalil Disrupts the Endocrine System in F1 Generation Mice. Mol. Cell. Endocrinol..

[50] Boberg J., Johansson H.K., Franssen D., Draskau M.K., Christiansen S., Cramer J., Pedersen M., Parent A.-S., Svingen T. (2023). Exposure to the Pesticides Linuron, Dimethomorph and Imazalil Alters Steroid Hormone Profiles and Gene Expression in Developing Rat Ovaries. Toxicol. Lett..

[51] Egbuta C., Lo J., Ghosh D. (2014). Mechanism of Inhibition of Estrogen Biosynthesis by Azole Fungicides. Endocrinology.

[52] Sciarra F., Campolo F., Franceschini E., Carlomagno F., Venneri M.A. (2023). Gender-Specific Impact of Sex Hormones on the Immune System. Int. J. Mol. Sci..

[53] Jin C., Luo T., Fu Z., Jin Y. (2018). Chronic Exposure of Mice to Low Doses of Imazalil Induces Hepatotoxicity at the Physiological, Biochemical, and Transcriptomic Levels. Environ. Toxicol..

[54] Jin C., Wang C., Zhang C., Ye Q., Jin Y., Shi L. (2024). Imazalil Resulted in Glucolipid Metabolism Disturbance and Abnormal m6A RNA Methylation in the Liver of Dam and Offspring Mice. Ecotoxicol. Environ. Saf..

[55] Roza M., Svanholm S., Berg C., Karlsson O. (2026). Juvenile Exposure to Imazalil Disrupts Epigenetic and Transcriptomic Regulation of Hepatic Energy Metabolism in *Xenopus tropicalis*. Sci. Total Environ..

[56] Xu M., Yu Y., Li J., Bi Y., Liu J., Fu C. (2025). A Mechanistic Perspective: How Does Tert-Butylhydroquinone Retain Antioxidative Efficacy in Edible Oils during Storage?. Food Chem..

[57] Khezerlou A., Pouya Akhlaghi A., Alizadeh A.M., Dehghan P., Maleki P. (2022). Alarming Impact of the Excessive Use of Tert-Butylhydroquinone in Food Products: A Narrative Review. Toxicol. Rep..

[58] 21 CFR 172.185—TBHQ. https://www.ecfr.gov/current/title-21/part-172/section-172.185.

[59] (2016). EFSA Panel on Food Additives and Nutrient Sources added to Food (ANS) Statement on the Refined Exposure Assessment of Tertiary-Butyl Hydroquinone (E 319). EFSA J..

[60] Whisel A.M., Rice C.D. (2025). Tert-Butylhydroquinone (TBHQ) Suppresses LPS- and Poly (I:C)-Induced RAW 264.7 Macrophage Activation Through Reduced NF-κB/Type 1 Interferon and Enhanced Antioxidant-Related Pathways. Toxics.

[61] Boss A., Freeborn R., Liu S., Zagorski J., Turley A., Gardner E., Rockwell C. (2019). The Food Additive tBHQ Impairs NK Cell Cytotoxicity against Influenza Infection (FS12-02-19). Curr. Dev. Nutr..

[62] Awali S., Rockwell C.E. (2025). The Nrf2 Activator, tBHQ, Blunts Dendritic Cell Function upon Influenza A Infection 9080. J. Immunol..

[63] Koh K., Cha Y., Kim S., Kim J. (2009). tBHQ Inhibits LPS-Induced Microglial Activation via Nrf2-Mediated Suppression of P38 Phosphorylation. Biochem. Biophys. Res. Commun..

[64] Saddawi-Konefka R., Seelige R., Gross E.T.E., Levy E., Searles S.C., Washington A., Santosa E.K., Liu B., O’Sullivan T.E., Harismendy O. (2016). Nrf2 Induces IL-17D to Mediate Tumor and Virus Surveillance. Cell Rep..

[65] Seelige R., Saddawi-Konefka R., Adams N.M., Picarda G., Sun J.C., Benedict C.A., Bui J.D. (2018). Interleukin-17D and Nrf2 Mediate Initial Innate Immune Cell Recruitment and Restrict MCMV Infection. Sci. Rep..

[66] Seelige R., Washington A., Bui J.D. (2017). The Ancient Cytokine IL-17D Is Regulated by Nrf2 and Mediates Tumor and Virus Surveillance. Cytokine.

[67] Kraft A.D., Johnson D.A., Johnson J.A. (2004). Nuclear Factor E2-Related Factor 2-Dependent Antioxidant Response Element Activation by Tert-Butylhydroquinone and Sulforaphane Occurring Preferentially in Astrocytes Conditions Neurons against Oxidative Insult. J. Neurosci..

[68] Imhoff B.R., Hansen J.M. (2010). Tert-Butylhydroquinone Induces Mitochondrial Oxidative Stress Causing Nrf2 Activation. Cell Biol. Toxicol..

[69] Jin Y., Boss A.P., Bursley J.K., Wilson C., Gangur V., Rockwell C.E. (2025). The Transcription Factor Nrf2 Links Th2-Mediated Experimental Allergy to Food Preservatives. Front. Immunol..

[70] Rockwell C.E., Zhang M., Fields P.E., Klaassen C.D. (2012). Th2 Skewing by Activation of Nrf2 in CD4+ T Cells. J. Immunol..

[71] Zagorski J.W., Turley A.E., Freeborn R.A., VanDenBerg K.R., Dover H.E., Kardell B.R., Liby K.T., Rockwell C.E. (2018). Differential Effects of the Nrf2 Activators tBHQ and CDDO-Im on the Early Events of T Cell Activation. Biochem. Pharmacol..

[72] van Esch G.J. (1986). Toxicology of *Tert*-Butylhydroquinone (TBHQ). Food Chem. Toxicol..

[73] National Toxicology Program (1997). NTP Toxicology and Carcinogenesis Studies of T-Butylhydroquinone (CAS No. 1948-33-0) in F344/N Rats and B6C3F(1) Mice (Feed Studies). Natl. Toxicol. Program. Tech. Rep. Ser..

[74] Németh K., Petschner P., Pálóczi K., Fekete N., Pállinger É., Buzás E.I., Tamási V. (2022). Chronic Exposure to the Food Additive tBHQ Modulates Expression of Genes Related to SARS-CoV-2 and Influenza Viruses. Life.

[75] Leonard E.R., Marques E.S., Roy M.A., Conlin S.M., Ranjan R., Timme-Laragy A.R. (2023). Dietary Exposure to the Food Preservative Tert-Butylhydroquinone (tBHQ) Impairs Zebrafish (Danio Rerio) Survival, Growth, Organ Development, and Gene Expression in Nrf2a-Dependent and Independent Ways. Food Chem. Toxicol..

[76] Freeborn R.A., Boss A.P., Liu S., Jin Y., Brocke S., Turley A.E., Zagorski J.W., Kennedy R.C., Gardner E.M., Rockwell C.E. (2019). The Immune Response to Influenza Is Suppressed by the Synthetic Food Additive and Nrf2 Activator, *Tert*-butylhydroquinone (tBHQ). FASEB J..

[77] Weiner M.L. (2014). Food Additive Carrageenan: Part II: A Critical Review of Carrageenan in Vivo Safety Studies. Crit. Rev. Toxicol..

[78] Rupert R., Rodrigues K.F., Thien V.Y., Yong W.T.L. (2022). Carrageenan From Kappaphycus Alvarezii (Rhodophyta, Solieriaceae): Metabolism, Structure, Production, and Application. Front. Plant Sci..

[79] Mendoza W., Ganzon-Fortes E., Villanueva R., Romero J., Montaño M.N. (2006). Tissue Age as a Factor Affecting Carrageenan Quantity and Quality in Farmed Kappaphycus Striatum (Schmitz) Doty Ex Silva. Bot. Mar..

[80] Álvarez-Viñas M., Souto S., Flórez-Fernández N., Torres M.D., Bandín I., Domínguez H. (2021). Antiviral Activity of Carrageenans and Processing Implications. Mar. Drugs.

[81] Chauhan P.S., Saxena A. (2016). Bacterial Carrageenases: An Overview of Production and Biotechnological Applications. 3 Biotech.

[82] Núñez-Santiago M.C., Tecante A., Garnier C., Doublier J.L. (2011). Rheology and Microstructure of κ-Carrageenan under Different Conformations Induced by Several Concentrations of Potassium Ion. Food Hydrocoll..

[83] Russo Spena S., Grizzuti N. (2025). κ-Carrageenan and Its Synergistic Blends: Next-Generation Food Gels. Gels.

[84] McKim J.M., Willoughby J.A., Blakemore W.R., Weiner M.L. (2019). Clarifying the Confusion between Poligeenan, Degraded Carrageenan, and Carrageenan: A Review of the Chemistry, Nomenclature, and in Vivo Toxicology by the Oral Route. Crit. Rev. Food Sci. Nutr..

[85] Shu Y., Liu X., Ma X., Gao J., He W., Cao X., Chen J. (2017). Immune Response Mechanism of Mouse Monocytes/Macrophages Treated with κ-Carrageenan Polysaccharide. Environ. Toxicol. Pharmacol..

[86] Cicinskas E., Begun M.A., Vikhareva V.V., Karetin Y.A., Kalitnik A.A. (2021). Immunological Effects of *Chondrus Armatus* Carrageenans and Their Low Molecular Weight Degradation Products. J. Biomed. Mater. Res..

[87] Sokolova E.V., Karetin Y., Davydova V.N., Byankina A.O., Kalitnik A.A., Bogdanovich L.N., Yermak I.M. (2016). Carrageenans Effect on Neutrophils Alone and in Combination with LPS *In Vitro*. J. Biomed. Mater. Res..

[88] Yuan H., Song J., Li X., Li N., Dai J. (2006). Immunomodulation and Antitumor Activity of κ-Carrageenan Oligosaccharides. Cancer Lett..

[89] Yuan H., Song J., Li X., Li N., Liu S. (2011). Enhanced Immunostimulatory and Antitumor Activity of Different Derivatives of κ-Carrageenan Oligosaccharides from Kappaphycus Striatum. J. Appl. Phycol..

[90] Villamil L., Infante Villamil S., Rozo G., Rojas J. (2019). Effect of Dietary Administration of Kappa Carrageenan Extracted from Hypnea Musciformis on Innate Immune Response, Growth, and Survival of Nile Tilapia (*Oreochromis niloticus*). Aquac. Int..

[91] Cheng A.-C., Chen Y.-Y., Chen J.-C. (2008). Dietary Administration of Sodium Alginate and κ-Carrageenan Enhances the Innate Immune Response of Brown-Marbled Grouper *Epinephelus fuscoguttatus* and Its Resistance against *Vibrio alginolyticus*. Vet. Immunol. Immunopathol..

[92] Fujiki K., Shin D.-H., Nakao M., Yanot T. (1997). Effects of κ-Carrageenan on the Non-Specific Defense System of Carp *Cyprinus carpio*. Fish. Sci..

[93] Mi Y., Chin Y.X., Cao W.X., Chang Y.G., Lim P.E., Xue C.H., Tang Q.J. (2020). Native κ-Carrageenan Induced-Colitis Is Related to Host Intestinal Microecology. Int. J. Biol. Macromol..

[94] Wu W., Zhen Z., Niu T., Zhu X., Gao Y., Yan J., Chen Y., Yan X., Chen H. (2017). κ-Carrageenan Enhances Lipopolysaccharide-Induced Interleukin-8 Secretion by Stimulating the Bcl10-NF-κB Pathway in HT-29 Cells and Aggravates *C. freundii*-Induced Inflammation in Mice. Mediat. Inflamm..

[95] Jiang H.-Y., Wang F., Chen H.-M., Yan X.-J. (2013). κ-Carrageenan Induces the Disruption of Intestinal Epithelial Caco-2 Monolayers by Promoting the Interaction between Intestinal Epithelial Cells and Immune Cells. Mol. Med. Rep..

[96] Wei W., Feng W., Xin G., Tingting N., Zhanghe Z., Haimin C., Xiaojun Y. (2016). Enhanced Effect of κ-Carrageenan on TNBS-Induced Inflammation in Mice. Int. Immunopharmacol..

[97] Wu W., Wang F., Gao X., Niu T., Zhu X., Yan X., Chen H. (2016). Synergistic Effect of κ-Carrageenan on Oxazolone-Induced Inflammation in BALB/c Mice. BMC Gastroenterol..

[98] Yin Y., Li M., Gu W., Zeng B., Liu W., Zhu L., Pi X., Primerano D.A., Yu H.D., Wei H. (2021). Carrageenan Oligosaccharides and Associated Carrageenan-Degrading Bacteria Induce Intestinal Inflammation in Germ-Free Mice. J. Genet. Genom..

[99] Shen S., Tong C., Li Z., Zhu Y., Hong T., Ni H., Jiang Z., Zheng M. (2026). Structural Characterization of κ-Carrageenan by Acetylation Modification and Its Effect on Intestinal Inflammation. Int. J. Biol. Macromol..

[100] Bhattacharyya S., Dudeja P.K., Tobacman J.K. (2008). Carrageenan-Induced NFκB Activation Depends on Distinct Pathways Mediated by Reactive Oxygen Species and Hsp27 or by Bcl10. Biochim. Biophys. Acta (BBA)-Gen. Subj..

[101] Bhattacharyya S., Dudeja P.K., Tobacman J.K. (2010). Tumor Necrosis Factor α-Induced Inflammation Is Increased but Apoptosis Is Inhibited by Common Food Additive Carrageenan. J. Biol. Chem..

[102] Ai L., Chung Y.-C., Lin S.-Y., Jeng K.-C.G., Lai P.F.-H., Xiong Z.-Q., Wang G. (2018). Carrageenan Polysaccharides and Oligosaccharides with Distinct Immunomodulatory Activities in Murine Microglia BV-2 Cells. Int. J. Biol. Macromol..

[103] Xu L., Yao Z., Wu H., Wang F., Zhang S. (2012). The Immune Regulation of κ-Carrageenan Oligosaccharide and Its Desulfated Derivatives on LPS-Activated Microglial Cells. Neurochem. Int..

[104] Yao Z., Xu L., Jin L., Wang T., Wang B., Li J., Bai Y., Wu H. (2022). κ-Carrageenan Oligosaccharides Induce Microglia Autophagy through AMPK/ULK1 Pathway to Regulate Their Immune Response. Int. J. Biol. Macromol..

[105] Relleve L., Abad L. (2015). Characterization and Antioxidant Properties of Alcoholic Extracts from Gamma Irradiated κ-Carrageenan. Radiat. Phys. Chem..

[106] Sun T., Tao H., Xie J., Zhang S., Xu X. (2010). Degradation and Antioxidant Activity of κ-Carrageenans. J. Appl. Polym. Sci..

[107] Weiner M.L., Nuber D., Blakemore W.R., Harriman J.F., Cohen S.M. (2007). A 90-Day Dietary Study on Kappa Carrageenan with Emphasis on the Gastrointestinal Tract. Food Chem. Toxicol..

[108] Lam C.S., O’Connell K., Monroy-Iglesias M.J., Du M., Romano M.E., Giovannucci E.L., Mao J.J., Kantor E.D. (2026). Evaluation of the Association Between Carrageenan and Systemic Inflammation: Results from the National Health and Nutrition Examination Survey. Mol. Nutr. Food Res..

[109] Borsani B., De Santis R., Perico V., Penagini F., Pendezza E., Dilillo D., Bosetti A., Zuccotti G.V., D’Auria E. (2021). The Role of Carrageenan in Inflammatory Bowel Diseases and Allergic Reactions: Where Do We Stand?. Nutrients.

[110] Kurihara K. (2015). Umami the Fifth Basic Taste: History of Studies on Receptor Mechanisms and Role as a Food Flavor. BioMed Res. Int..

[111] Chow J.Y., Shahar S., Haron H., Ong Y.Q. (2025). A Convenience Sample Based Market Survey of the Food Additive Monosodium Glutamate (MSG) in Processed Foods in Malaysia. J. Nutr. Sci..

[112] Bellisle F., Monneuse M.O., Chabert M., Larue-Achagiotis C., Lanteaume M.T., Louis-Sylvestre J. (1991). Monosodium Glutamate as a Palatability Enhancer in the European Diet. Physiol. Behav..

[113] Yamaguchi S., Takahashi C. (1984). Interactions of Monosodium Glutamate and Sodium Chloride on Saltiness and Palatability of a Clear Soup. J. Food Sci..

[114] Mortensen A., Aguilar F., Crebelli R., Di Domenico A., Dusemund B., Frutos M.J., Galtier P., Gott D., Gundert-Remy U., EFSA Panel on Food Additives and Nutrient Sources Added to Food (ANS) (2017). Re-Evaluation of Glutamic Acid (E 620), Sodium Glutamate (E 621), Potassium Glutamate (E 622), Calcium Glutamate (E 623), Ammonium Glutamate (E 624) and Magnesium Glutamate (E 625) as Food Additives. EFSA J..

[115] Yu H., Wang R., Zhao Y., Song Y., Sui H., Wu Y., Miao H., Lyu B. (2023). Monosodium Glutamate Intake and Risk Assessment in China Nationwide, and a Comparative Analysis Worldwide. Nutrients.

[116] Tian W., Yong L., Miao H., Yang X., Wei Q., Wei S., Sui H., Xiao X., Li J., Wang H. (2025). Analysis of Glutamate Content in Food and Dietary Exposure Assessment to Glutamate among the Chinese Population. Appl. Food Res..

[117] Zanfirescu A., Ungurianu A., Tsatsakis A.M., Nițulescu G.M., Kouretas D., Veskoukis A., Tsoukalas D., Engin A.B., Aschner M., Margină D. (2019). A Review of the Alleged Health Hazards of Monosodium Glutamate. Compr. Rev. Food Sci. Food Saf..

[118] Sakai R., Motoi R., Amino Y., Hayamizu K. (2026). Concomitant Food Intake Markedly Alters Plasma Glutamic Acid Kinetics after Oral Monosodium Glutamate Administration in Rats: Relevance to Dietary Safety Evaluation. Toxicol. Rep..

[119] Hassan Z.A., Arafa M.H., Soliman W.I., Atteia H.H., Al-Saeed H.F. (2014). The Effects of Monosodium Glutamate on Thymic and Splenic Immune Functions and Role of Recovery (Biochemical and Histological Study). J. Cytol. Histol..

[120] Das D., Banerjee A., Bhattacharjee A., Mukherjee S., Maji B.K. (2022). Dietary Food Additive Monosodium Glutamate with or without High-Lipid Diet Induces Spleen Anomaly: A Mechanistic Approach on Rat Model. Open Life Sci..

[121] Xu J., Tang M., Liu Y., Xu J., Xu X. (2022). Safety Assessment of Monosodium Glutamate Based on Intestinal Function and Flora in Mice. Food Sci. Hum. Wellness.

[122] Peng Q., Huo D., Ma C., Jiang S., Wang L., Zhang J. (2018). Monosodium Glutamate Induces Limited Modulation in Gut Microbiota. J. Funct. Foods.

[123] Zhao B., Babayev H., Zeyneloglu C., Pat Y., Yazici D., Ardicli S., García-Sánchez A., Viscardi O.G., Akdis M., Nadeau K.C. (2025). Monosodium Glutamate Induces Cellular Stress, Endoplasmic Reticulum Stress, Mitochondrial Dysfunction, and Cell Death in Intestinal Epithelial Cells. Allergy.

[124] Su Q., Yang L., Xiao J., Cao H., Wang H. (2025). MYR Ameliorated MSG-Induced Immune Dysfunction in HFD Mice *via* Modulation of Gut Microbiota and SCFAs Metabolism. Food Biosci..

[125] Neves D., Salazar I.L., Almeida R.D., Silva R.M. (2023). Molecular mechanisms of ischemia and glutamate excitotoxicity. Life Sci..

[126] Meldrum B.S. (2000). Glutamate as a Neurotransmitter in the Brain: Review of Physiology and Pathology. J. Nutr..

[127] Rivera-Carvantes M.C., Jarero-Basulto J.J., Feria-Velasco A.I., Beas-Zárate C., Navarro-Meza M., González-López M.B., Gudiño-Cabrera G., García-Rodríguez J.C. (2017). Changes in the Expression Level of MAPK Pathway Components Induced by Monosodium Glutamate-Administration Produce Neuronal Death in the Hippocampus from Neonatal Rats. Neuroscience.

[128] Abdou H.M., Hamaad F.A., Elmageed G.M.A., Katano H., Ghoneum M.H. (2025). Efficacy of Plasmalogens on Monosodium Glutamate-Induced Neurotoxicity in Male Rats Through NF-κB and P38 MAPK Signaling Pathways. Oxid. Med. Cell. Longev..

[129] Firgany A.E.-D.L., Sarhan N.R. (2020). Quercetin Mitigates Monosodium Glutamate-Induced Excitotoxicity of the Spinal Cord Motoneurons in Aged Rats via P38 MAPK Inhibition. Acta Histochem..

[130] Özkan S.Ç., Toraman E. (2025). Tannic Acid Protection against Oxidative and Inflammatory Effects of Monosodium Glutamate on Testis: Molecular and Biochemical Approach. Reprod. Biol..

[131] Umukoro S., Oluwole G.O., Olamijowon H.E., Omogbiya A.I., Eduviere A.T. (2015). Effect of Monosodium Glutamate on Behavioral Phenotypes, Biomarkers of Oxidative Stress in Brain Tissues and Liver Enzymes in Mice. WJNS.

[132] Okwudiri O., Sylvanus A.C., Peace I.A. (2012). Monosodium Glutamate Induces Oxidative Stress and Affects Glucose Metabolism in the Kidney of Rats. IJBCRR.

[133] Hong H., Kim B.S., Im H.-I. (2016). Pathophysiological Role of Neuroinflammation in Neurodegenerative Diseases and Psychiatric Disorders. Int. Neurourol. J..

[134] Sarkar S. (2022). Microglial Ion Channels: Key Players in Non-Cell Autonomous Neurodegeneration. Neurobiol. Dis..

[135] Hassan A.H., El Nashar E.M., Al-Zahrani N.S., Zafrah H., El Henafy H.M. (2026). Maternal Monosodium Glutamate Exposure Disrupts Leptin and Insulin Signaling in the Hypothalamus, Activating NF-κB and mTOR Inflammatory Pathways, Contributing to Metabolic Dysfunction in Male Offspring: Adipocyte and Cell Biology. Int. J. Obes..

[136] Hasenböhler A., Javaux G., Payen de la Garanderie M., Szabo de Edelenyi F., Agaësse C., De Sa A., Deschasaux-Tanguy M., Fezeu L., Srour B., Touvier M. (2024). Food Additive Monosodium Glutamate and Risk of Cardiovascular Diseases-NutriNet-Santé Cohort. Eur. J. Public Health.

[137] Lisiecka M.Z. (2026). The Role of Monosodium Glutamate in Food Allergies and Its Health Implications. J. Am. Nutr. Assoc..

[138] Gao W., Guo L., Yang Y., Wang Y., Xia S., Gong H., Zhang B.-K., Yan M. (2022). Dissecting the Crosstalk between Nrf2 and NF-κB Response Pathways in Drug-Induced Toxicity. Front. Cell Dev. Biol..

[139] McKim J.M., Baas H., Rice G.P., Willoughby J.A., Weiner M.L., Blakemore W. (2016). Effects of Carrageenan on Cell Permeability, Cytotoxicity, and Cytokine Gene Expression in Human Intestinal and Hepatic Cell Lines. Food Chem. Toxicol..

[140] Sun N., Pat Y., Babayev H., Ardicli S., Ogulur I., Bu X., Li M., Jia S., Rückert B., Dhir R. (2026). Effects of Common Food Additives Kappa-, Iota- and Lambda-Carrageenans on Intestinal Epithelial Cell Activation and Barrier Disruption. Clin. Exp. Allergy.

[141] Rusyn I., Chiu W.A., Wright F.A. (2022). Model Systems and Organisms for Addressing Inter- and Intra-Species Variability in Risk Assessment. Regul. Toxicol. Pharmacol..

[142] Van Norman G.A. (2019). Limitations of Animal Studies for Predicting Toxicity in Clinical Trials. JACC Basic Transl. Sci..

[143] Trede N.S., Langenau D.M., Traver D., Look A.T., Zon L.I. (2004). The Use of Zebrafish to Understand Immunity. Immunity.

[144] Brugman S. (2016). The Zebrafish as a Model to Study Intestinal Inflammation. Dev. Comp. Immunol..

[145] Hartung T. (2018). Perspectives on *In Vitro* to *In Vivo* Extrapolations. Appl. In Vitro Toxicol..

[146] Macko P., Palosaari T., Whelan M. (2021). Extrapolating from Acute to Chronic Toxicity in Vitro. Toxicol. In Vitro.

[147] Han P., Li X., Yang J., Zhang Y., Chen J. (2024). Advancing Toxicity Predictions: A Review on in Vitro to in Vivo Extrapolation in Next-Generation Risk Assessment. Env. Health.

[148] Landsiedel R., Birk B., Demuth P., Fabian E., Hewitt N.J., Hollnagel H.M., Scheel J. (2024). The Use of Toxicokinetic Information for Setting Concentrations of In Vitro Toxicity Tests and for Interpreting Their Results: A Proposed Workflow. Appl. In Vitro Toxicol..

[149] Lynch C., Sakamuru S., Ooka M., Huang R., Klumpp-Thomas C., Shinn P., Gerhold D., Rossoshek A., Michael S., Casey W. (2024). High-Throughput Screening to Advance In Vitro Toxicology: Accomplishments, Challenges, and Future Directions. Annu. Rev. Pharmacol. Toxicol..

[150] Ledwith R., Stobernack T., Bergert A., Bahl A., Pink M., Haase A., Dumit V.I. (2024). Towards Characterization of Cell Culture Conditions for Reliable Proteomic Analysis: In Vitro Studies on A549, Differentiated THP-1, and NR8383 Cell Lines. Arch. Toxicol..

[151] Merly L., Smith S.L. (2017). Murine RAW 264.7 Cell Line as an Immune Target: Are We Missing Something?. Immunopharmacol. Immunotoxicol..

[152] Tahiri M., Johnsrud C., Steffensen I.-L. (2023). Evidence and Hypotheses on Adverse Effects of the Food Additives Carrageenan (E 407)/Processed Eucheuma Seaweed (E 407a) and Carboxymethylcellulose (E 466) on the Intestines: A Scoping Review. Crit. Rev. Toxicol..

[153] Castle L., Andreassen M., Aquilina G., Bastos M.L., Boon P., Fallico B., FitzGerald R., Frutos Fernandez M.J., Grasl-Kraupp B., EFSA Panel on Food Additives and Flavourings (FAF) (2026). Guidance on the Scientific Data Requirements for an Application for Authorisation of a Food Additive Submitted under Regulation (EC) No 1331/2008. EFS2.

[154] Buonsenso F. (2025). Scientific and Regulatory Perspectives on Chemical Risk Assessment of Pesticides in the European Union. J. Xenobiotics.

[155] Vandenberg L.N., Colborn T., Hayes T.B., Heindel J.J., Jacobs D.R., Lee D.-H., Shioda T., Soto A.M., Vom Saal F.S., Welshons W.V. (2012). Hormones and Endocrine-Disrupting Chemicals: Low-Dose Effects and Nonmonotonic Dose Responses. Endocr. Rev..

[156] Pekmezci H., Sipahi S., Başaran B. (2025). Health Risk Assessment of Dietary Chemical Exposures: A Comprehensive Review. Foods.

[157] Ullah S., Ali S., Atif M., Ullah M., Nabi G., Freire L.G.D., Kamimura E.S., Nejad A.S.M., de Oliveira C.A.F. (2025). The Potential Influence of Food Additives and Contaminants on the Gut Microbiota: A Comprehensive Review. Food Chem. Toxicol..

[158] Li P., Qu R., Li M., Sheng P., Jin L., Huang X., Xu Z.Z. (2024). Impacts of Food Additives on Gut Microbiota and Host Health. Food Res. Int..

[159] Cerk K., Aguilera-Gómez M. (2022). Microbiota Analysis for Risk Assessment: Evaluation of Hazardous Dietary Substances and Its Potential Role on the Gut Microbiome Variability and Dysbiosis. EFSA J..

[160] Garrido-Romero M., Pazos F., Sánchez-Martínez E., Benito C., Gómez-Ruiz J.Á., Borrego-Yaniz G., Bowes C., Broll H., Caminero A., Caro E. (2024). Relevance of Gut Microbiome Research in Food Safety Assessment. Gut Microbes.

[161] Nair A.B., Jacob S. (2016). A Simple Practice Guide for Dose Conversion between Animals and Human. J. Basic Clin. Pharm..

[162] International Programme on Chemical Safety (2010). Characterization and Application of Physiologically Based Pharmacokinetic Models in Risk Assessment.

[163] Groothuis F.A., Heringa M.B., Nicol B., Hermens J.L.M., Blaauboer B.J., Kramer N.I. (2015). Dose Metric Considerations in *in Vitro* Assays to Improve Quantitative *In Vitro*–*In Vivo* Dose Extrapolations. Toxicology.

[164] Fountoucidou P., Veskoukis A.S., Kerasioti E., Docea A.O., Taitzoglou I.A., Liesivuori J., Tsatsakis A., Kouretas D. (2019). A Mixture of Routinely Encountered Xenobiotics Induces Both Redox Adaptations and Perturbations in Blood and Tissues of Rats after a Long-Term Low-Dose Exposure Regimen: The Time and Dose Issue. Toxicol. Lett..

[165] Kraemer M.V.d.S., Fernandes A.C., Chaddad M.C.C., Uggioni P.L., Rodrigues V.M., Bernardo G.L., Proença R.P.d.C. (2022). Food Additives in Childhood: A Review on Consumption and Health Consequences. Rev. Saúde Pública.

[166] Witkowski M., Grajeta H., Gomułka K. (2022). Hypersensitivity Reactions to Food Additives—Preservatives, Antioxidants, Flavor Enhancers. Int. J. Environ. Res. Public Health.

[167] Shaw I.C. (2014). Chemical Residues, Food Additives and Natural Toxicants in Food—The Cocktail Effect. Int. J. Food Sci. Tech..

[168] Besedin D., Van T.T.H., Besedin J., Krohn C., Shah R., Eri R. (2026). The Combined Effect of Eight Common Food Additives on the Human Gut Microbiota. Food Chem. Toxicol..

[169] Wang X., Fan D., Yang Y., Gimple R.C., Zhou S. (2023). Integrative Multi-Omics Approaches to Explore Immune Cell Functions: Challenges and Opportunities. iScience.

[170] Karczewski K.J., Snyder M.P. (2018). Integrative Omics for Health and Disease. Nat. Rev. Genet..

[171] Vinken M. (2013). The Adverse Outcome Pathway Concept: A Pragmatic Tool in Toxicology. Toxicology.

