# Supplementary material for: Food-Associated Xenobiotics and Innate Immunity: Molecular and Cellular Effects of Imazalil, TBHQ (Tert-Butylhydroquinone), Carrageenans, and Monosodium Glutamate
Source: Int J Mol Sci. 2026 Oct 26;27(21):9580. doi: 10.3390/ijms27219580 (PMC13763563; doi:10.3390/ijms27219580)
Supplement: Supplementary file 1 [file ijms-27-09580-s001.zip › ijms-4596338-supplementary.pdf]

Table S1. Study-level evidence on innate immune responses to imazalil: experimental models, material characteristics, exposure conditions, findings, and relevance to human dietary exposure.

Study	Model and material	Dose and delivery	Duration / challenge	Measured findings	Interpretation and exposure relevance
Jin et al., 2016 [11]	Male ICR mice; 42 total; 5 weeks old at purchase Imazalil; ≥97% purity; acetone used for chow preparation and evaporated before feeding	25, 50, 100 mg/kg bw/day Incorporated into chow; food available at night	28 days; control/high-dose follow-up 35 days without IMZ	Colonic inflammation and microbiota changes; cytokine transcripts and fecal lipocalin-2 assessed.	Mucosal inflammatory evidence; microbial changes do not establish impaired leukocyte function. Administered doses are not food-residue concentrations.
Jin et al., 2021 [12]	C57BL/6 dams; 10/group; male/female F1 sampled at 20 days and 7 weeks; F2 at 20 days Imazalil; ≥97% purity	0.025‰ and 0.25‰; authors estimated approximately 5 and 50 mg/kg bw/day Maternal oral exposure; precise delivery technique not specified in exposure subsection	From conception through lactation; offspring unexposed after weaning; F1 females bred at 9 weeks	Altered mucus, epithelial defense-related transcripts, bile acids and IL-22 expression across examined generations.	Multigenerational observations; gestational exposure can also expose F1 and developing F2 germ cells. No demonstrated transgenerational immune inheritance.
Jin et al., 2018 [13]	Male C57BL/6 mice; 5 weeks old at purchase; 1-week acclimation Imazalil; purity ≥97%	0.1, 0.5, 2.5 mg/kg bw/day Oral; dissolved in pure water; delivery technique not explicitly identified	2, 5 or 15 weeks; control/high-dose recovery for 45 days	Microbiota, mucus and epithelial transporter changes; recovery differed between intestinal regions.	Longer oral exposure is informative, but mucus/transporter measurements do not directly establish permeability or infection resistance.
Ratajczak-Wrona et al., 2026 [14]	Human blood from 15 healthy male donors, 23–32 years; assay-specific PMN, buffy-coat or purified-neutrophil preparations Imazalil	0.07, 0.7, 7 ng/mL Direct *ex vivo* exposure; NO arm in RPMI with 4% newborn calf serum	Chemotaxis: 60-min pretreatment + 60-min migration; uptake: 60-min pretreatment + 60-min particle incubation; NO/viability: 2 h	Latex uptake decreased at 0.07 and 0.7 ng/mL; chemotaxis decreased at 0.7 ng/mL. Spontaneous NBT reduction increased at 0.7 ng/mL. Viability remained approximately 98% after 2 h.	Direct functional evidence; no microbial-killing or in vivo infection assay. Lowest concentration linked by authors to a prior serum measurement, not established typical intake.

Abbreviations: bw, body weight; WT, wild type.

Table S2. Study-level evidence on innate immune responses to TBHQ: experimental models, material characteristics, exposure conditions, findings, and relevance to human dietary exposure.

Study	Model and material	Dose and delivery	Duration / challenge	Measured findings	Interpretation and exposure relevance
Boss et al., 2018 [15]	Female C57BL/6J mouse splenocytes; gated NK cells TBHQ	1 and 5 μM Direct exposure; ethanol vehicle 0.01%	30-min pretreatment; PMA/ionomycin stimulation for 24 h	Reduced CD25/CD69 induction and IFN-γ, granzyme B and perforin; altered maturation phenotype.	Activation and effector-associated readouts; not a direct target-cell-killing assay. Mixed-cell culture; Nrf2 necessity not established by these observations.
Whisel and Rice, 2025 [60]	RAW 264.7 murine macrophages TBHQ	0.1, 1, 5 μM Direct cell exposure; LPS 0.1 μg/mL or poly(I:C) 20 μg/mL	4-h pretreatment; 24-h functional assays; transcriptomic arm uses 12-h challenge	0.1 μM increased particle uptake; 5 μM reduced NO/IL-10. IL-6 responses depended on dose and stimulus. MTT used for concentration selection.	Functional uptake plus mediator/transcriptomic evidence. Not uniform phagocytic suppression; antioxidant gene induction does not prove Nrf2 dependence.
Boss et al., 2019 [61]	Female C57BL/6 mice; influenza A/PR/8/34 TBHQ in feed	0.0014% in diet Dietary feeding; intranasal infection	2 weeks before infection; assessment 2 days after infection	Reduced NK granzyme B reported; CD25/CD69 not affected in abstract.	Preliminary dietary infection-model evidence. Granzyme B is not direct cytotoxic killing; do not import 1% diet from Scopus AI overview.
Awali and Rockwell, 2025 [62]	Female C57BL/6 mice; lung and mediastinal lymph-node dendritic cells TBHQ in AIN-93G diet	0.0014% in AIN-93G diet Dietary feeding; influenza A/PR/8/34 challenge	2 weeks before infection; post-infection sampling time unspecified in accessed abstract	Reduced MHC II, CD40/CD80/CD86, IL-6 and IL-12 reported in conference abstract.	Preliminary phenotype/mediator evidence; not proof of impaired antigen presentation or dietary human risk.
Koh et al., 2009 [63]	BV-2 and primary cortical microglia; neuronal conditioned-medium assay TBHQ	BV-2: 10, 20, 30 μM; primary microglia: 20 μM; LPS 10 ng/mL Direct exposure; Nrf2 shRNA intervention in BV-2 cells	BV-2: 12-h pretreatment, then LPS 12 h (cytokines) or 48 h (NO/PGE2). Primary microglia: 24-h pretreatment, then LPS 24–48 h	Reduced inflammatory mediators; Nrf2 knockdown partly attenuated protection. Conditioned medium reduced neuronal toxicity.	Mechanistic innate-cell evidence with pathway intervention. Neuroinflammatory model; no dietary exposure inference.

Seelige et al., 2018 [65]	C57BL/6-background WT and gene-deficient mouse models; MCMV TBHQ	50 mM TBHQ/DMSO injection solution; administered mass pending Intraperitoneal; PBS/DMSO control	Recruitment/mediator readouts at 24 h; infection-arm dosing schedule pending	Innate-cell recruitment and viral burden assessed; TBHQ reduced susceptibility to MCMV.	Host-defense evidence in a pharmacological, non-dietary setting. Separate genetic axis findings from the complete set of TBHQ effects.

Abbreviations: bw, body weight; WT, wild type.

Table S3. Study-level evidence on innate immune responses to carrageenans: experimental models, material characteristics, exposure conditions, findings, and relevance to human dietary exposure.

Study	Model and material	Dose and delivery	Duration / challenge	Measured findings	Interpretation and exposure relevance
Bhattacharyya et al., 2010 [17]	NCM460 human colonic epithelial cells λ-, κ-, κ8 (eight-disaccharide κ fragment) and ι-carrageenan; linkage-specific enzyme pretreatment	1 mg/L (=1 μg/mL) Direct exposure; with/without enzymatic predigestion	24-h cell exposure; predigestion 24 h	Enzymatic cleavage changed IL-8/BCL10 responses; ROS response was not altered by the tested cleavage treatments.	Supports structural specificity rather than a universal low-molecular-weight toxicity rule.
Wu et al., 2022 [18]	Male C57BL/6 and germ-free mice; C. rodentium challenge Repurified κ-carrageenan from *Eucheuma cottonii*; mean Mw 198 kDa; sulfate 20.15%; starting product purity >90%	1.7, 8.3, 41.7 mg/kg; pathogen 10^9 CFU/mouse Oral gavage; saline control	90-day carrageenan exposure; infection outcomes 7 days after challenge	Reduced SCFAs/mucus thickness without marked inflammation alone; infection worsened outcomes. Fecal transfer supported microbial contribution.	Challenge-dependent susceptibility and microbiota intervention are informative; not proof of spontaneous human disease.
Bhattacharyya et al., 2008 [19]	NCM460 human colonic epithelial cells; additional mechanistic arms λ-Carrageenan in the identified TLR4-blocking experiment	1 and 10 μg/mL in TLR4-blocking arm Direct exposure; blocking-antibody pretreatment	2-h antibody pretreatment; 24-h carrageenan exposure	λ-Carrageenan increased IL-8/BCL10-associated responses; TLR4 blocking attenuated the response.	Epithelial innate signaling; not a leukocyte functional or dietary-risk assay.
Shu et al., 2017 [85]	RAW 264.7 murine macrophages κ-Carrageenan: title calls it polysaccharide, Methods calls it oligosaccharide (98% purity); molecular weight unspecified	MTT series: 25–400 μg/mL; core mediator/uptake experiments: 100 μg/mL Direct exposure	Core exposure 24 h; neutral-red incubation 30 min; TLR4 inhibitor pretreatment 6 h	Increased MTT signal, neutral-red uptake, NO and TNF-α; TLR4 inhibitor attenuated TNF-α response.	Neutral-red uptake does not establish microbial engulfment or killing. Ambiguous material identity prevents confident classification as native food-grade carrageenan.

Cicinskas et al., 2021 [86]; *in vitro* arm	Murine peritoneal macrophages C. armatus κ (174.5 kDa), λ (260–300 kDa); κ acid-hydrolysis products 0.7–20 kDa and λ autohydrolysis products 10–170 kDa	Migration: 100 μg/mL; phagocytosis: 1, 10, 100 μg/mL Direct exposure	Migration 24 h; uptake: 3-h pretreatment + 1.5-h pHrodo E. coli particle incubation	Most preparations increased motility and reduced phagocytosis; λ degradation products reduced motility without affecting phagocytosis.	Direct functional, preparation-dependent evidence. Neither native nor degraded material has a uniform direction of effect.
Cicinskas et al., 2021 [86]; feeding arm	Mice; peritoneal lavage/macrophage assays; initially 10/group C. armatus κ (174.5 kDa), λ (260–300 kDa); κ acid-hydrolysis products 0.7–20 kDa and λ autohydrolysis products 10–170 kDa	100 μg/kg bw/day Oral gavage in saline; volume 25 μL/g/day	7 days; collection on day 8	Reduced particle uptake across preparations; degradation products increased motility. κ degradation products reduced leukocyte count. No treatment-attributed gross organ changes.	Do not transfer *in vitro* direction or concentration to the oral arm.
Sokolova et al., 2016 [87]	Healthy human donor blood; neutrophil/monocyte suspension after erythrocyte lysis; PBS without protein κ 200 kDa; λ 200 kDa; κ/β 420 kDa; acid-degraded κ/β 1.9 kDa	12.5, 25, 50, 100 μg/mL; with/without LPS Direct exposure, with/without LPS	Opsonized latex uptake 30 min; ROS/MPO 1 h; whole-blood NO derivatives 20 h	Preparation-dependent latex uptake and ROS/MPO responses; carrageenans could attenuate LPS-induced ROS/MPO. NO derivatives were not induced.	Direct phagocyte measurements; particle uptake and MPO do not establish microbial killing or complete phagosome maturation. No equivalence to dietary exposure.
Mi et al., 2020 [93]; water arm	Male C57BL/6J mice; low-fat diet (10%) versus high-fat diet (45%); HFD established for 10 weeks Extracted native κ-carrageenan from *Kappaphycus alvarezii*; mean Mw 365 kDa; sulfate 11.02%	0.5% in drinking water Drinking water	6-week carrageenan exposure	High-fat context: increased DAI, colonic MPO and TLR4 mRNA. Low-fat context: no colitis signs reported.	Oral exposure; vehicle and background diet modify outcome. Not interchangeable with incorporation in feed.

Mi et al., 2020 [93]; feed arm	Male C57BL/6J mice; low-fat diet (10%) versus high-fat diet (45%); HFD established for 10 weeks<br>Extracted native κ-carrageenan from *Kappaphycus alvarezii*; mean Mw 365 kDa; sulfate 11.02%	5% incorporated into diet<br>Dietary feeding	6-week carrageenan exposure	No visible inflammatory effects reported for supplemented diet, in contrast to high-fat/water arm.	Retain the null outcome; dietary route alone does not establish customary human exposure.
C10<br>Weiner et al., 2007 [107]	Fischer 344 rats; 20/sex/group<br>κ-Carrageenan; Mw 196–257 kDa; low-MW tail 1.9–12% below 50 kDa	0, 25000, 50000 ppm in diet<br>Dietary incorporation	90 days	No treatment-related adverse effects in assessed general-toxicology endpoints; gastrointestinal assessment included.	Characterized feeding study provides null evidence for measured outcomes, not exclusion of unmeasured specialized immune effects.
C12<br>Lam et al., 2026 [108]	NHANES adults: primary n=922; secondary n=622<br>Carrageenan-containing protein beverages; subtype not assigned	Exposure proxy; no administered dose<br>Habitual beverage consumption	Cross-sectional survey; not an intervention duration	No overall association with higher CRP; subgroup findings were hypothesis-generating.	Human systemic biomarker association, not causal or functional immune evidence; proxy does not quantify total carrageenan intake.
C11<br>McKim et al., 2016 [139]	HT-29/HCT-8 intestinal cells; separate Caco-2 absorption arm<br>Characterized carrageenan preparations; κ/λ/ι mapping pending	HT-29/HCT-8: 0.1, 1, 10 μg/mL; Caco-2: 100, 500, 1000 μg/mL<br>Direct exposure	24 h for cytokine assays; absorption timing pending	No cytokine induction/cytotoxicity in intestinal lines; no detectable carrageenan permeability or cytotoxicity in absorption model.	Important null cellular evidence. Lack of polymer absorption is not the same endpoint as preserved barrier resistance.

Abbreviations: bw, body weight; DAI, disease activity index; MPO, myeloperoxidase; SCFAs, short-chain fatty acids. WT, wild type.

Table S4. Study-level evidence on innate immune responses to monosodium glutamate: experimental models, material characteristics, exposure conditions, findings, and relevance to human dietary exposure.

Study	Model and material	Dose and delivery	Duration / challenge	Measured findings	Interpretation and exposure relevance
Motwadie et al., 2021 [20]	Adult male Wistar rats; 40 total across four groups<br>MSG; other additives studied in separate groups	20 mg/kg bw/day<br>Intragastric gavage; freshly prepared suspension; distilled-water control	90 consecutive days	Leukocyte/spleen and cytokine changes. Author-labelled "phagocytic activity" was unchanged, but assessed by colorimetric NBT reduction.	NBT is a redox-related surrogate, not direct particle uptake or microbial killing. Methods name neutrophils; Results call the cells macrophages. Retain this reporting inconsistency.
Das et al., 2024 [21]	Rat thymus/spleen model (v7)<br>MSG, ≥99% purity in primary methods excerpt	600 mg/kg bw/day<br>Gavage needle	28 days	Redox/inflammatory signaling and immune-organ changes; attenuation with quercetin.	Repeated bolus exposure and immune-organ responses; pleiotropic rescue does not prove a uniquely necessary pathway.
Hassan et al., 2014 [119]	Adult male rats<br>MSG	3 g/kg bw/day<br>Oral gavage	8 weeks; recovery group a further 4 weeks	Thymic/splenic biochemical and histological abnormalities; partial recovery described.	High-dose repeated bolus; substantial adaptive/organ-level contribution. No direct host-defense test.
Das et al., 2022 [120]	Male adult Wistar rats; 24 total, 8/group<br>MSG alone or with high-lipid mixture	600 mg/kg bw/day; lipid mixture 10 mL/kg/day<br>Oral; aqueous MSG; exact administration technique pending	4 weeks	Splenic abnormalities under MSG alone or combined dietary/metabolic conditions.	In vivo organ-level study, not isolated immune-cell incubation. Cannot infer phagocytic killing from splenic injury.
Xu et al., 2022 [121]	Male BALB/c mice; 4 weeks old at purchase; 12/group<br>MSG	30, 300, 1500 mg/kg bw/day; NaCl comparator 100 mg/kg/day<br>Oral gavage; pure-water control	Once daily for 5 weeks	30 mg/kg: no significant changes in assessed CRP, intestinal cytokines, sIgA or fecal albumin; 1500 mg/kg: intestinal structural/microbial changes.	Dose-specific intestinal evidence; no measured clinical cardiovascular disease or universal immune-safety threshold.
Peng et al., 2018 [122]	Human participants; n=12<br>MSG	2 g/day (v7)<br>Oral consumption; matrix pending	4 weeks	Limited microbiota compositional/functional modulation.	Human microbiome evidence; no basis for claiming preserved or impaired innate-cell function.

Zhao et al., 2025 [123]	Caco-2 monolayers and gut-on-chip epithelial models MSG	0.25%, 0.5%, 1% MSG; percentage basis still requires Methods supplement Direct epithelial exposure	TEER: 1–2 days, with short-term measurements to 4 h; ROS: 24 h; flux: 3 days	TEER decreased at 1% after 1 day and at 0.25%/0.5% after 2 days; 1% increased flux after 3 days and ROS after 24 h. Cytotoxicity occurred from 1%.	Direct barrier injury model; cytotoxicity limits immune-specific attribution and intake extrapolation.

Abbreviations: bw, body weight; DAI, disease activity index; MPO, myeloperoxidase; SCFAs, short-chain fatty acids. WT, wild type.
